# *Purriato* is a conserved small open reading frame gene that interacts with the CASA pathway to regulate muscle homeostasis and epithelial tissue growth in *Drosophila*

**DOI:** 10.3389/fcell.2023.1117454

**Published:** 2023-03-10

**Authors:** Jose I. Pueyo, Jorge Salazar, Carolina Grincho, Jimena Berni, Benjamin P. Towler, Sarah F. Newbury

**Affiliations:** ^1^ Brighton and Sussex Medical School, University of Sussex, Brighton, United Kingdom; ^2^ Department of Biochemistry and Biomedicine, School of Life Sciences, University of Sussex, Brighton, United Kingdom

**Keywords:** smORF peptides, sarcomerogenesis, cell proliferation, constitutive assisted selective autophagy (CASA), proteostasis, wing imaginal disc, *Drosophila*

## Abstract

Recent advances in proteogenomic techniques and bioinformatic pipelines have permitted the detection of thousands of translated small Open Reading Frames (smORFs), which contain less than 100 codons, in eukaryotic genomes. Hundreds of these actively translated smORFs display conserved sequence, structure and evolutionary signatures indicating that the translated peptides could fulfil important biological roles. Despite their abundance, only tens of smORF genes have been fully characterised; these act mainly as regulators of canonical proteins involved in essential cellular processes. Importantly, some of these smORFs display conserved functions with their mutations being associated with pathogenesis. Thus, investigating smORF roles in *Drosophila* will not only expand our understanding of their functions but it may have an impact in human health. Here we describe the function of a novel and essential *Drosophila* smORF gene named *purriato* (*prto*). *prto* belongs to an ancient gene family whose members have expanded throughout the Protostomia clade. *prto* encodes a transmembrane peptide which is localized in endo-lysosomes and perinuclear and plasma membranes. *prto* is dynamically expressed in mesodermal tissues and imaginal discs. Targeted *prto* knockdown (KD) in these organs results in changes in nuclear morphology and endo-lysosomal distributions correlating with the loss of sarcomeric homeostasis in muscles and reduction of mitosis in wing discs. Consequently, *prto* KD mutants display severe reduction of motility, and shorter wings. Finally, our genetic interaction experiments show that *prto* function is closely associated to the CASA pathway, a conserved mechanism involved in turnover of mis-folded proteins and linked to muscle dystrophies and neurodegenerative diseases. Thus, this study shows the relevance of smORFs in regulating important cellular functions and supports the systematic characterisation of this class of genes to understand their functions and evolution.

## Introduction

Decoding the essential information stored within the genome is key to understanding the function of biological organisms, their origins and evolution. The rapid developments in sequencing technologies and bioinformatic approaches have increased our knowledge of the great diversity of functional genes contained within genomes. Comparative analyses have significantly enriched the annotation of genomic “canonical” functional genes (i.e., gene, transcript, coding sequence (CDS)) and have also revealed the existing genetic variation in our genomes (Roy et al., 2010). Despite the usefulness of current genome annotations in linking genomic functions (genotype) to phenotypes, as well as mapping genetic diseases, there is a hidden genome which is under-represented in these annotations. These hidden genes comprise thousands of putatively functional small Open Reading Frames (smORFs).

smORFs are defined as stretches of DNA between start and stop codons which potentially encode peptides of less than 100 amino-acids. Despite the existence of hundreds of thousands of smORFs in eukaryotic genomes, their functional importance has been overlooked due to the vast number of “junk” smORFs which are non-functional (Basrai et al., 1997; Couso and Patraquim, 2017). Therefore, the challenge lies in the differentiation between coding and non-coding smORFs. Previous genome annotation algorithms dismissed smORFs due to their small length, often regarding them as meaningless as they are unable to obtain the high conservation scores (i.e., BLAST) that are an indicator of functionality (Ladoukakis et al., 2011; Landry et al., 2015; Mackowiak et al., 2015). In addition, the detection of smORF encoded peptides by mass spectrometry is challenging experimentally due to their small size and bioinformatically due to the lack of smORFs annotations in databases used for their identification (Saghatelian and Couso, 2015). Therefore, to overcome these challenges, specific experimental and bioinformatics approaches are being used to assess smORF expression and functionality in different species (Frith et al., 2006; Kastenmayer et al., 2006; Hanada et al., 2007; Ladoukakis et al., 2011; Mackowiak et al., 2015). These studies show that there are a substantial number of putatively functional smORFs in eukaryotes, with an overall prediction that smORFs could increase the number of new functional genes in eukaryotic genomes by up to 5%.

Ribosome profiling (Ribo-Seq) is a technique that isolates mRNA fragments protected by the ribosome and prepares these fragments for the construction of libraries for NextGen sequencing. Comparison between the ribosome footprints (Ribo-Seq) and mRNA abundance (RNA-Seq) from the same sample enables genome-wide quantitative and qualitative measurements of translation, which has revolutionized our understanding of ORFeome (Ingolia et al., 2009; Ingolia et al., 2012). Ribo-seq studies have revealed pervasive translation of smORFs in metazoan genomes, identifying thousands of smORFs present in alternate frames in canonical CDSs, in untranslated regions (UTRs), and long non-coding RNAs (Ingolia et al., 2012; Lee et al., 2012; Aspden et al., 2014; Bazzini et al., 2014; Ji et al., 2015; Raj et al., 2016; Patraquim et al., 2020; Wright et al., 2022). Despite the large numbers of translated smORFs, only a relatively small number have been fully characterised. These show a wide variety of molecular functions, such as ligands for cellular receptors, signalling modulators, ionic pump inhibitors and translation regulators (Galindo et al., 2007; Kondo et al., 2010; Magny et al., 2013; Pauli et al., 2014; Rathore et al., 2018). Notably, recent genome-wide functional screens in cultured cells have demonstrated that translated smORFs are indeed required for cell growth and viability (Chen et al., 2020; Prensner et al., 2021; Na et al., 2022). However, the lack of annotation of smORFs in the genome, due to differences in experimental and bioinformatic approaches in these studies, plus the relatively small number of translated smORF being characterised, has prevented a more comprehensive understanding of their physiological, cellular and molecular roles.

Comparative analyses of translation properties and molecular and evolutionary signatures of these translated smORFs have revealed the existence of distinct classes of smORFs which could represent different stages of smORF functionalization (Aspden et al., 2014; Couso and Patraquim, 2017; Patraquim et al., 2022). This classification suggested the existence of five different ORF classes, which displayed different size, conservation, organisation, translation, structure, and amino acid preference that each different class would represent a step in gene, protein and peptide evolution. These distinct classes describe global properties that are conserved through different metazoan genomes and display an array of functions (Couso and Patraquim, 2017). For instance, lncORFs and upstream ORFs (uORFs) both have a very similar median ORF size (22-24 codons), and they are found in polycistronic arrangements within the transcript. Generally, the conservation scores of these two ORF classes are low, their codon usages are not random but are different from canonical proteins, and despite being translated their translational efficiency is low in comparison with canonical proteins (Aspden et al., 2014; Couso and Patraquim, 2017; Patraquim et al., 2020; Patraquim et al., 2022). Although, several uORFs and lncORFs encode peptides with important cellular functions (Magny et al., 2013; Anderson Douglas et al., 2015; Matsumoto et al., 2017; Jayaram et al., 2021) the majority of uORF and lncORF classes appear to function as translational regulators of main ORFs and in a variety of non-coding regulatory functions respectively (Aspden et al., 2014; Couso and Patraquim, 2017; Patraquim et al., 2020; Patraquim et al., 2022).

One of the other relevant smORF classes is the *short CDS* (*shCDS*) class*. shCDSs* encode for peptides that are longer in size (median 80 aa) and generally have an alpha helix transmembrane domain (Aspden et al., 2014; Couso and Patraquim, 2017). More than 800 of these *shCDSs* are actively translated in *Drosophila* (Aspden et al., 2014; Patraquim et al., 2020). Strikingly, similar number of *shCDSs* have been found in other eukaryotes displaying conserved sequence, structure and evolutionary signatures, suggesting that they could fulfil important biological roles (Pueyo et al., 2016a; Couso and Patraquim, 2017). Despite their abundance, only tens of *shCDS* genes have been characterised in *Drosophila.* These studies have shown that shCDS peptides mainly interact with canonical proteins and regulate their functions by diverse mechanisms (Pueyo et al., 2016a; Plaza et al., 2017), playing essential roles in signalling processes, organelle homeostasis and cytoskeleton dynamics (Pueyo et al., 2016b; Ding et al., 2017; Johnson et al., 2021; Magny et al., 2021). Importantly, many *shCDS* functions are conserved in humans and their mutations associated with pathogenesis (Pueyo et al., 2016a; Patraquim et al., 2020). Thus, investigating novel *shCDS* roles in *Drosophila* will not only expand our understanding regarding their functional repertoire but it is likely to improve our knowledge of human health.

In this paper, we have characterised the function of a *shCDS* gene named *purriato* (*prto*) that encodes for a single transmembrane peptide. *prto* is a member of an ancient gene family whose members are conserved throughout the Protostomia clade. Our functional characterisation in *Drosophila* shows *prto* is an essential gene, and its knock down by RNAi results in a reduction of the size of proliferative organs, muscle dysfunction and premature death. Our phenotypic analysis and genetic interactions show that *prto* is involved in regulating the turnover of sarcomeric proteins by interacting with the chaperone assisted selective autophagy (CASA) pathway in muscle cells. Importantly, the knockdown of CASA members produce *prtoKD-like* wing phenotypes, which are further enhanced by removing *prto* gene function. Thus, the role of Prto in CASA mediated protein turnover appears to be broad and crucial in the regulation of different cellular processes showing a novel and essential role of *prto* in the regulation of the conserved CASA pathway. Since mutations of CASA members in humans are linked to proteostaxis related diseases, such as muscular dystrophies, cardiomyopathies and neuropathies (Sarparanta et al., 2020), this study can pave the way for the identification of Prto-like candidates in humans to modulate the CASA pathway for therapeutic purposes.

## Materials and methods

### Fly husbandry

*Drosophila melanogaster* stocks were obtained from the Bloomington (BL) or the Vienna (VDRC) *Drosophila* stock centers. The following Gal4 drivers for targeted expression were used: Mef2Gal4, 24BGal4, Dlmo-Gal4 (MS1096 BL:8860), 69B-Gal4 (BL:1774), en-Gal4 (BL:30557;30564). The following RNAi lines were used to knock down gene expression: UAS-prto-RNAi (VDRC: v30420), UAS-prto-RNAi#2 (BL:53700), UAS-GFP-RNAi (BL:9330), UAS-stv-RNAi (BL:42564), UAS-NUAK-RNAi (BL:31885), UAS-cher-RNAi (BL:26307), UAS-Hsc70-4 RNAi (BL:54810), UAS-atg8a RNAi (BL:28989). The UAS-Dcr stocks (BL:24650; BL:24651) were used to increase the RNAi knock-down efficiency. The FUCCI stock to label cell cycle progression was used (BL:55100). The G203-GFP; Sls-GFP, UAS-Cher-90-GFP stocks were kindly gifted by M. Landgraf and J. Ylänne respectively. In addition, stv^1^ and NUAK null alleles and UAS-stv-V5 stocks were kindly provided by E. Geisbrecht. The UAS-prto-FL and UAS-GFP-prto and UAS-Flag-prto transgenic flies were newly generated (see below).

Fly crosses were grown in standard cornmeal medium at 25°C but L1 larvae offspring were also shifted to other temperatures such as 18°C and 29°C as stated for controlling the UAS-construct expression level. For climbing assays crosses were kept at 25°C and flies were raised in rich molasses medium to stimulate the number of individuals reaching adulthood.

### Antibodies and fluorescent markers

The following primary antibodies were used: mouse anti-GFP (1:500; Roche), rabbit anti-GFP (1:400; Molecular Probes), mouse anti-FLAG (1:500; Sigma), mouse anti-Kettin/Sls (1:10; Developmental Studies Hybridoma Bank [DSHB]), mouse anti-Laminin (1:20; DSHB), rabbit anti-active Caspase3 (1:1000; BioLabs), mouse anti-Phosphohistone H3 (1:300; Cell Signalling). Secondary Donkey anti-mouse Alexa 488 and anti-rabbit-Alexa 555 conjugated antibodies were used (1:400; Molecular Probes) and Donkey anti-mouse Rhodamine and anti-rabbit-FITC antibodies were used (1:200; Jackson ImmunoResearch). Rhodamine and Cy5-congugated phalloidin were used to label actin filaments (1:50; Molecular Probes). DAPI (Sigma) was utilized for revealing nuclei.

### Immunocytochemistry

Late 3^rd^ instar wandering larvae were pinned and then filleted in a 3% agar plate using surgical scissors and then fixed with 4% Paraformaldehyde in PBS for 20 min. Body wall muscles were rinsed 3 times with PBT (PBS with 0.5% Tween) and then blocked with PBT with 2% BSA (Sigma) and 10% horse serum (Vector Laboratories) for 1 h at room temperature. Primary antibodies were incubated overnight at 4°C in blocking buffer. Samples were then washed 3 times with PBT then incubated with secondary antibodies in blocking buffer for 2 h at room temperature or 4°C overnight. The secondary antibodies were then removed, and samples washed 3 times with PBT and 3 times with PBS. Samples were then incubated with fluorescent conjugated Phalloidin and DAPI for 45 min at room temperature to reveal the actin cytoskeleton and myonuclei. Dissection and staining of adult indirect thoracic flight muscles were carried out as described in Magny et al. (2013). Immunocytochemistry of imaginal discs was performed following the protocols described in Galindo et al. (2007).

### *In situ* hybridisation

Embryos staged at 0–22 h AEL were collected and fixed by shaking in 4% PFA: Heptane (1:1) fixative for 20 min. Fixed embryos were washed in PBT (1x PBS and 0.1% Tween) five times, and then incubated in 2.5 µL of proteinase K diluted in 1 mL of PBT with rotation for 2 min. Embryos were then washed twice with 2 mg/ml glycine in PBT followed by several PBT washes and incubation with rotation for 10 min in hybridization solution (HS) diluted 1:1 in PBT. Samples were then pre-hybridized for 1–2 h in 1 mL HS at 55°C followed by hybridisation with the selected probes at 50 ng of labelled probe in 200 µL of HS at 55°C overnight without rotation. After the overnight incubation and post hybridisation washes, embryos were then blocked with 0.1% BSA in PBT for 30 min followed by incubation in a dilution 1:2000 of mouse anti-Dig antibody (Roche) in PBT-0.1% BSA with shaking for 3–4 h. Detection of transcripts was achieved by using a substrate solution (9 µL NBT and 7 µL BCIP in 2 mL of Genius 3 buffer (100 mM Tris-HCl (pH 9.5), 10 mM NaCl, 50 mM MgCl_2_) in the dark at room RT. For imaginal disc and larval muscles, protocol used was described in Galindo et al. (2007), and Magny et al. (2013).

### S2 cell culture, transfection and immunocytochemistry

S2 cells were cultured in 10% FBS (v/v) Schneider’s media at 25°C to a confluent stage. Transfections and staining protocols were carried out as described in Pueyo et al. (2016b). We used primary rabbit and mouse anti-Flag antibody (1:500; Sigma) and secondary Donkey anti-mouse FITC (1:1000; Jackson ImmunoResearch), anti-mouse Rhodamine (1:400;Jackson ImmunoResearch) and anti-rabbit Cy5 (1:400; Jackson ImmunoResearch) for detection of tagged-smORF peptides. For detection of mitochondria S2 cells were incubated in 500 nM Mitotracker Red CMXRos (Life Technologies) for 45 min and then fixed for immunohistochemistry. For Lysosome colocalization experiments, cells were incubated in Lysotracker-DND99 (1:1000; Molecular Probes) for 15 min, then mounted and observed *in vivo*. Images of 5–10 cells per transfection culture were captured on the LSM510 Axioskop 2 or on the Leica TCS SP8 confocal microscopes. For colocalization analysis Z-stack images were taken and analysed with the ImageJ plugin “Mander’s Coefficients” which was used to calculate Pearson’s correlation coefficient of tag to tag signal in 2 different channels.

### Generation of transgenic flies

Extraction of RNA from S2 cells was achieved using manufacturer’s TRIZOL RNA protocol, and subsequently a cDNA library was generated using random primers and Moloney Murine Leukemia Virus Reverse Transcriptase Kit (Invitrogen). Amplification of the full length Prto cDNA was achieved by PCR (Qiagen), using the forward primer 5′ GAG​AGA​GAA​TTC​CTC​TAA​TCA​GCT​GTT​TGG​TTT​GT 3′ and the reverse primer 5′ CAC​ACA​CTC​GAG​ATT​TGG​ATG​TTT​GTT​TTA​CTT​AGC 3′ containing EcoRI and XhoI restriction sites respectively. Restriction sites were used for subsequent cloning of the Prto PCR product in the pUASt vector. Similarly, Prto ORF fragments for N-terminal and C-terminal tagging were amplified as above using the following primers: Ct-forward 5′CAC​CCT​CTA​ATC​AGC​TGT​TTG​GTT​TGT, and Ct-reverse 5′ GTC​ATC​CTC​GTC​GTT​GCG​CAC; Nt-forward 5′ CAC​CTC​CGC​ATC​CGC​TGC​CCG​A and Nt-reverse 5′ ACA​TTT​GGA​TGT​TTG​TTT​TAC​TTA​GCT​T. PCR products were cloned in the pENTR™ Directional TOPO® Cloning vector (Invitrogen). Integration of Prto ORF fragments to final destination vectors (T. Murphy, unpublished results; obtained from the *Drosophila* Genomics Research Center) was achieved by LR recombination following the Gateway system (Invitrogen). UAS-prto constructs were verified by sequencing and injected to generate transgenic flies (BestGene).

### Phenotypic analysis

Quantification of muscle defect in L2 and L3 whole larvae was achieved by isolating larvae expressing GFP trapped protein labelling the Z-disc in control or *prto* KD. Larvae were drowned in 70% ethanol for 1 h and mounted in glycerol for visualisation. Images were captured using the fluorescent Zeiss microscope and Zen 2.6 software. Muscle phenotypes were categorised as truncated or thin muscles.

Pupal case phenotypes were analysed by calculating the axial ratio which is the length of the pupae divided by the width as described in Brooks et al. (2020).

Adult male and female wings were dissected, mounted separately, and the wing size was measured by calculating the area (total wing or per wing compartment) using the “outline” tool of the Zen 2.6 software. The number of wing cells were quantified by counting number of trichomes in predetermined square in different wing areas and calculate the average of trichomes and then extrapolate trichome number to the whole wing area as described in Towler et al. (2020).

Rapid iterative negative geotaxis (RING) method, which is based on the fly’s innate escape response so they climb up the walls of the tube after being tapped to its bottom, was used to assess locomotor defects in adult flies (Gargano et al., 2005). Young flies (1–2 days as adults) were separated by sex and genotype and put in groups of 10 in different tubes marked at 4 and 8 cm height. Negative geotaxis and recording of flies climbing (3 repeats) was then performed accounting for what percentage were able to climb and/or reach the marks at 5, 10, 15 and 30 s (*n* = 30 per genotype and sex).

At each trial of locomotion behaviour, third-instar larvae were washed to remove traces of food and transferred to a 240 × 240 mm^2^ arena coated with a 2 mm thick layer of 0.8% agar, where they crawled freely for 5 min. After acclimatization, movies were recorded for 10 min at 25°C illumination with Infrared light following the Frustrated Total Internal Reflection (FTIR)-based imaging method (Risse et al., 2017). Movies were recorded with a Basler acA2040-180 km CMOS camera at 2048 × 2048 px^2^ resolution and 2 frames per second (Sims et al., 2019), using Pylon and StreamPix software, mounted with a 16 mm KOWA IJM3sHC. SW VIS-NIR Lens and 825 nm high performance long pass filter (Schneider, IF-093). The momentum x:y coordinates for each larva was obtained with FIM track (Risse et al., 2017) and used to calculate the average velocity.

For survival assays standard vials containing cornmeal, sucrose and yeast medium were used. 10 recently emerged flies (males or females) were placed in each vial and kept in an incubator at 25°C and checked the percentage of flies alive every 2 days for 14 days. Survival assays were performed in triplicate per genotype and sex.

The mitotic index was calculated by the ratio of cells going through mitosis (H3P positive cells) in the posterior compartment (en-Gal4;UAS-dsRed positive cells) and the H3P positive cells in the anterior compartment (ds-Red negative cells) in control and experimental conditions (Towler et al., 2020).

### RNA extraction and qRT-PCR

RNA was extracted from wandering L3 larval carcases aged with a 3 h egg lay using a standard phenol-chloroform protocol. Larvae were homogenised in QIAzol lysis reagent (Qiagen), mixed with 0.2 volume chloroform and spun at 12,000 g for 15 min. The aqueous phase was precipitated in 1 volume 100% isopropanol and RNA was pelleted at 15,000 g for 30 min. The RNA pellet was washed twice in 80% ethanol before drying and resuspension in H_2_O. DNase treatment was performed using the Turbo DNA-free kit (Invitrogen) following manufacturer’s instructions. RNA concentration and purity was assessed on a Nanodrop One.

1 µg of RNA for each sample was reverse transcribed using the High Capacity cDNA Reverse Transcription kit (Applied Biosystems). A no RT control was performed in parallel. qRT-PCR was performed using the following primers: *prto* Fw: 5′-GCT​GTT​TGG​TTT​GTT​CGT​TGT​G-3′, *prto* Rev: 5′-ATG​TCC​GGA​ATC​TCG​TTC​CA-3′, *rpl32* Fw: 5′-TAA​GCT​GTC​GCA​CAA​ATG​GC-3′, *rpl32* Rev: 5′-TCG​ACA​ATC​TCC​TTG​CGC​TT-3′ which were optimised to give 99.01% and 92.6% efficiency for *prto* and *rpl32* respectively. qRT-PCR was performed using the PowerTrack SYBR Green Master Mix (Applied Biosystems) in technical triplicate including a no template control. Cycling conditions were as follows: 95°C 5s followed by 58°C 30s cycled 40 times. Melt curve analysis was performed following manufacturer’s instructions. Data analysis was performed using the ∆∆ct method with *rpl32* as a reference gene.

### Phylogenetic analyses

Clustal W (DNASTAR) and MAFFT (http://mafft.cbrc.jp/aligment/server) (Katoh et al., 2019) were used to align Prto peptide sequences (L-INS-I MAFFT alignment was used with scoring matrix BLOSUM62; gap opening penalty 1.53). For alignment confidence scores and extraction of well-aligned residues the Guidance 2 package was used (http://guidance.tau.ac.il) (Sela et al., 2015). The Phylo.io tools within MAFFT package was used for constructing Neighbor-Joining tree using WAG model, Alpha ∞, Bootstrap resampling = 100 (Robinson et al., 2016).

### Statistical analysis

All statistical analyses were performed in GraphPad Prism 5 (GraphPad Software. Inc., La Jolla, CA). T-tests were used to compare the means of single test groups to single control groups. If multiple comparisons were required, a one-way ANOVA was performed with a post-test to compare the means of each pair of samples.

## Results

### Identification and characterisation of the *purriato* gene

To investigate the cellular functions of translated smORFs, we focused on the *shCDS* class, as they are generally highly translated, accrue high conservation scores, and present peptide structures which are generally good indicators of functionality. The *CG9034* gene was chosen for detailed characterisation from the shCDS’s pool because its function is unknown, and therefore we named it *purriato (prto)*, which is an invented word with no meaning. The *prto* gene is located in the X chromosome and has 2 annotated transcripts differing in their 5′UTRs which encode a unique peptide (Gramates et al., 2022) (Supplementary Figure S1A). Prto peptide appears to be conserved in other insects, is highly expressed in specific tissues and produced abnormal phenotypes in RNAi genome-wide screens (Schmidt et al., 2012; Gramates et al., 2022).

The *prto* gene displays a dynamic temporal expression profile, with its mRNA expression levels varying from very high to moderately high during embryogenesis and throughout larval, pupal and adult stages (Brown et al., 2014). In addition, *prto* is significantly expressed in specific organs and tissues, such as in the adult carcass, imaginal discs, head or testes (Brown et al., 2014).

**FIGURE 1:**
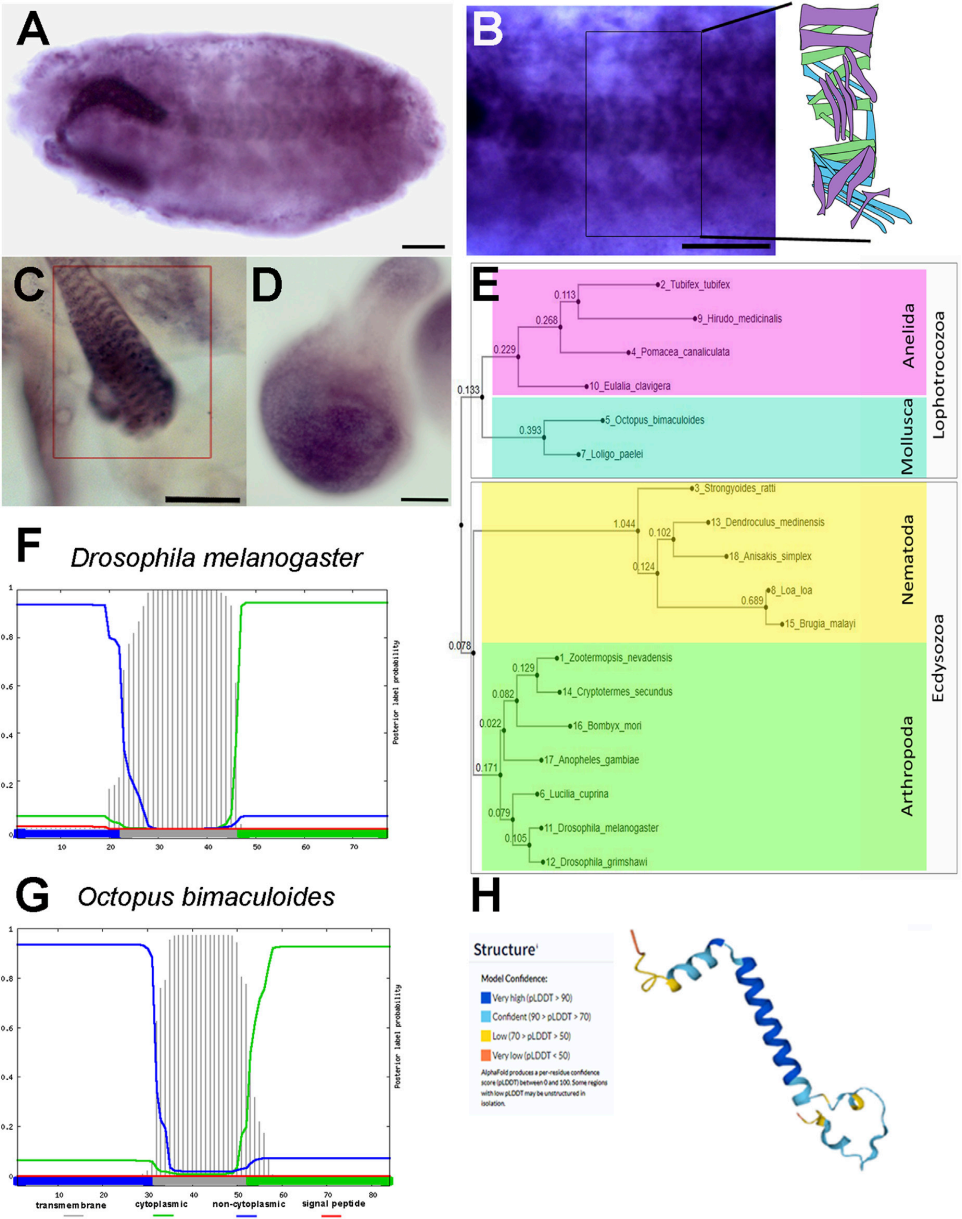
*prto* pattern of expression, and its protein sequence and structure conservation. **(A–D)** *In situ* hybridisation using digoxigenin-labelled *prto* probe. **(A)** Stage 17 embryo showing labelling in the somatic muscles. **(B)** High magnification of B showing the stereotypical array of somatic muscles per hemisegment as shown in diagram in inset. **(C)** L3 larval somatic muscle showing *prto* mRNA expression in the sarcolemma (rectangle). **(D)** *prto* mRNA expression in the pouch of the 96hr AEL wing imaginal disc. **(E)** Neighbor-Joining phylogenetic tree showing the evolutionary distances of Prto peptides across Protostomes. **(F,G)** Secondary structure predictions of signal peptide and transmembrane domains in Prto peptides by Phobius program from *Drosophila melanogaster* **(F)** and *Octopus bimaculoides* **(G)** showing a conserved single transmembrane topology (grey bars). **(H)** 3D predicted structure of *Drosophila* Prto peptide using the Alphafold program, displaying in blue very high confidence of helical structure. Scale bars denote 50 µm.

To better characterise the developmental and spatial profile of *prto* mRNA we performed *in situ* hybridisations in various organs and different stages. During early embryogenesis, *prto* mRNAs are maternally provided and distributed ubiquitously (stage 3; Supplementary Figure S1C). Slightly later in development, *prto* transcripts are restricted to the cephalic furrow (arrowhead) and the mesoderm when it begins to form (stage 6; Supplementary Figure S1D). Importantly, *prto* mesodermal expression is maintained through mesoderm development and differentiation in embryonic somatic and visceral muscles (Figures 1A, B; Supplementary Figures S1D–F) and larval muscles (Figure 1C). In addition, we have found that *prto* is highly expressed in the pouch of the wing imaginal discs (Figure 1D), which is supported by the modENCODE transcriptome data (Brown et al., 2014) and wing disc single cell transcriptomics (Li et al., 2022).

We also investigated *prto* expression profile at the translation level. Poly-Ribo-Seq data showed high levels of productive *prto* translation in S2 cells (Supplementary Figure S1B) (Aspden et al., 2014) and during embryonic stages (Patraquim et al., 2020). These translation profiles are in accordance with Prto peptide levels identified by mass spectrometry in embryogenesis (Casas-Vila et al., 2017). In addition, the *prto* translation has also been reported in larval muscles (Chen and Dickman, 2017). Thus, these temporal and spatial profiles at the transcriptional and translational levels provide the developmental framework to explore the putative roles of Prto peptides at the molecular and cellular levels.

### *Purriato* encodes a conserved single transmembrane peptide located in specific intracellular compartments

The *Drosophila prto* gene encodes a 77 amino-acid peptide with a predicted secondary structure containing an alpha-helix transmembrane domain according to JNet and Phobius algorithms (Käll et al., 2004; Drozdetskiy et al., 2015) (Supplementary Figure S2A; Figure 1F). In addition, the confidence of the 3D model for Prto is very high supporting that a single transmembrane Prto structure is likely (Figure 1H) (Jumper et al., 2021).

Next, we assessed the evolutionary conservation of Prto by performing PSI-BLAST searches (Altschul et al., 1997; Johnson et al., 2008) and pairwise sequence comparisons to identify Prto homologues using MAFFT and Clustal W (Thompson et al., 1994; Sela et al., 2015; Katoh et al., 2019). Our analysis revealed that Prto peptides are conserved among Protostomia. Prto homologues are found in other arthropods, such as insects (Figure 1E; Supplementary Figure S2C). In addition, we identified Prto homologue peptides in other Ecdysozoans such as Nematoda, and Lophotrochozoans such as Annelida and Mollusca. Our searches did not identify Prto in Deuterostomia (Figure 1E; Supplementary Figures S2B, C). Importantly, the predicted Prto peptide secondary structure obtained using the Phobius program (Madeira et al., 2022) seems to be conserved throughout the Prto peptide family (Figures 1F, G). In addition, striking conservation in stretches of amino-acids at the carboxyl-terminus (Ct) were also observed outside the transmembrane domain (Supplementary Figures S2B, C). Thus, this level of amino-acid and structure conservation strongly suggests that transmembrane Prto peptides could have a role in membrane-related cellular processes.

To corroborate the predicted transmembrane structure of Prto peptides and further explore their cellular localisation we generated different N-terminus and C-terminus tagged Prto constructs and expressed them in *Drosophila* S2 cells. These differentially tagged Prto peptides showed strong co-localization to intracellular membrane compartments with a Pearson’s correlation coefficient (R) of 0.86 for Prto-Venus vs. FLAG-Prto and 0.87 for GFP-Prto vs. Prto-FLAG (Supplementary Figures S2D–D″, E). Next, to identify the membrane compartments to which Prto peptides localises, we repeated the staining using known cellular markers. This revealed that GFP-Prto peptides are highly localised in endosomes displaying a Pearson’s coefficient of 0.87 with the Hemotin peptide (Supplementary Figures S2F–F″, I) (Pueyo et al., 2016b). Reduced co-localisation of GFP-Prto peptides was found with mitochondria (Mitotracker; R = 5.5; Supplementary Figures S2G–G″, I) and lysosomes (Lysotracker; R = 5.7; Supplementary Figures S2H–H″, I). Thus, the deep conservation of Prto transmembrane peptides and their co-localization with endosomes, lysosomes and mitochondria suggest that function in these intracellular organelles.

### *Purriato* is required for locomotion and proper muscle contraction

Several genome-wide RNAi screens have reported that *CG9034* knockdown (KD) produce generic defective phenotypes in specific fly organs (Schmidt et al., 2012). For instance, *CG9034* KD using the *Mef2* (muscle) and *Pannier* (thorax) Gal4 drivers led to loss of motility (Schnorrer et al., 2010), and shorter bristles respectively (Mummery-Widmer et al., 2009), but further characterisation have not been attempted.

**FIGURE 2:**
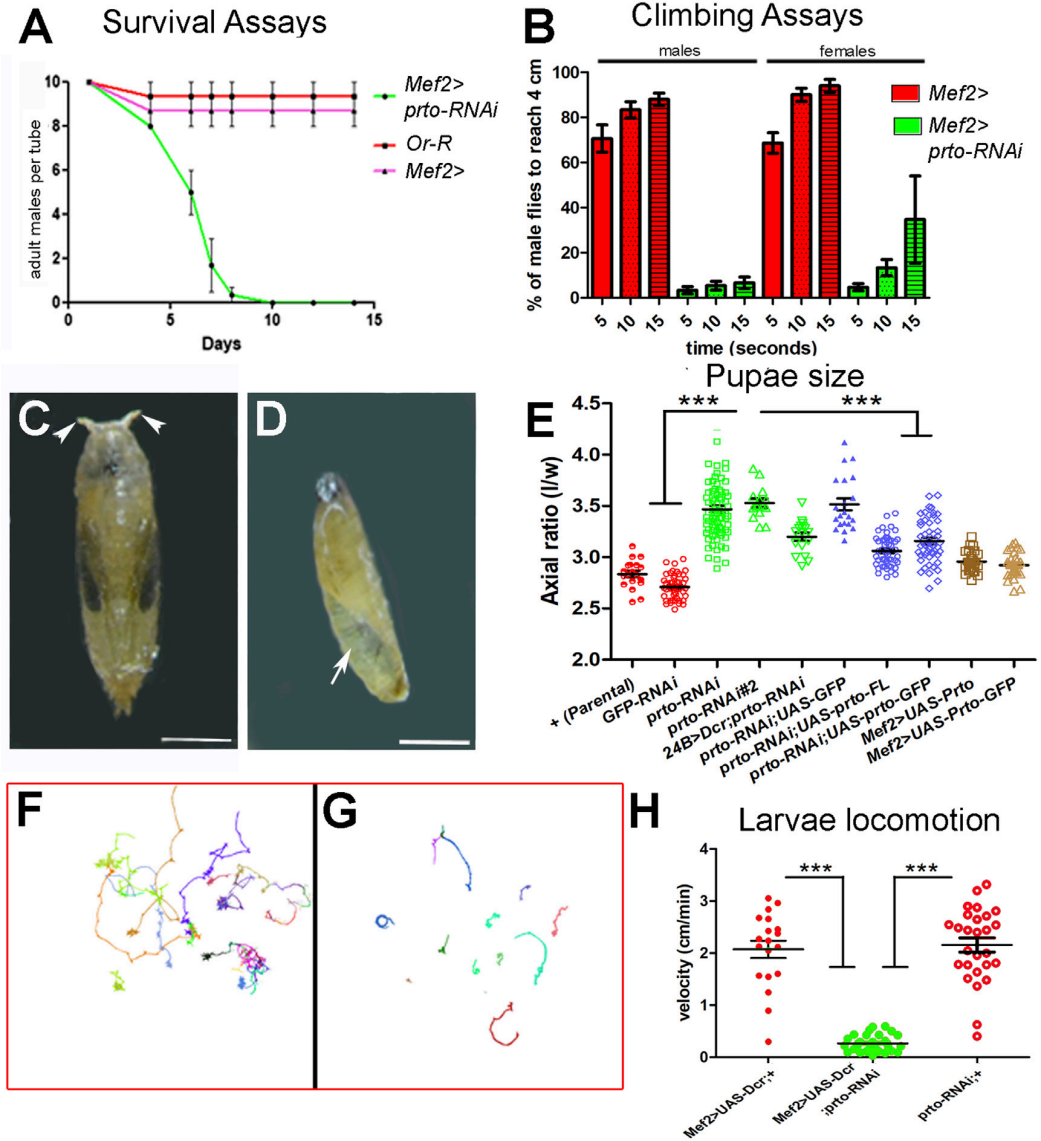
*prto* knock-down phenotypic analyses. **(A)** Survival assays of male adult flies showing a striking reduction of viability in *Mef2>prto-RNAi* males in comparison to parental *Mef2*> control and wild-type *Or-R* males at 29°C. *n* = 10 per replica, 3 replicas, error bars represent 95% CI. **(B)** Climbing analysis shows that both *Mef2>prto-RNAi* male and female flies have a reduced locomotion ability on reaching 4 cm mark in geotaxis experiments in comparison to controls (29°C). 1way ANOVA with Bonferroni post test statistical analysis was carried out. *n* = 15 per replica, error bars represent 95% CI. **(C,D)** Pupal cases of control **(C)** and *Mef2>UAS-Dcr;prto-RNAi* **(D)** at 29°C. Note that *prto* KD pupae are smaller in size, rarely differentiate adult structures, retain air bubble (arrow) and they lack anterior spiracles (arrowheads). Scale bar 1 mm. **(E)** Axial ratio measurements (length/width) of pupal case of controls (red), *prto-RNAi* KDs (green), rescue genotypes (blue) showing *prto* specificity of phenotype and ectopic *prto* full-length and GFP-tagged expression (brown) in the muscles. All knockdown, rescue and overexpression lines were driven with Mef2-Gal4 unless otherwise indicated 1 way ANOVA with Bonferroni post test statistical analysis. *n* = 15–77, error bars represent 95% CI, *** = *p* < 0.0001. **(F,G)** Locomotion behaviour of *Mef2>UAS-Dcr*; + **(F)** and *Mef2>UAS-Dcr; prto-RNAi* **(G)** L3 larvae raised at 29°C showing reduced locomotion in *prto* KDs. **(H)** Velocity measurements from exploratory behaviours show that *Mef2>UAS-Dcr;prto-RNAi* L3 larvae move more slowly than parental *Mef2>Dcr;+ and prto-RNAi* controls. 1way ANOVA with a Tukey’s post test statistical analysis. *n* = 19–31, error bars represent 95% CI, *** = *p* < 0.0001.

With previous data suggesting a role of *prto* in muscles, we set out to perform a detailed characterisation of *prto* function. Specific knockdown in muscles was achieved through driving *prto-RNAi* (GD4003) with the *Mef2Gal4* (Mef2>) driver at 29°C, which resulted in some lethality in late pupal stages, but escapers were able to produce adult offspring. Female and male *prto* KD adult flies were short lived (Figure 2A). Over time, surviving flies developed a held up wing posture (not shown) and display climbing defects in comparison with controls in negative geotaxis (Gargano et al., 2005) with males showing more severe phenotypes (Figure 2B). These results supported previous published observations (Schnorrer et al., 2010) and thus validating our functional approach. To understand the *prto* KD progressive deterioration in wing posture the thoracic indirect flight muscles (IFM) were phenotypically analysed. *prto-RNAi* KD myofibrils were slightly disorganised and displayed faded actin filaments in sarcomeres in comparison to controls (Supplementary Figures 3A, B). Although there were no major defects in the number of these myofibrils. Therefore, our results suggest that *prto* could be important in the regulation of physiological muscle processes.

To further characterise the *prto* function in muscles we crossed the *prto-RNAi* lines with lines expressing *Dicer*2 gene (*Dcr*) to stimulate RNAi silencing and enhance the knockdown (Dietzl et al., 2007). Expression of *prto-RNAi* using *Mef2>UAS-Dcr* driver at 29°C decreased *prto* mRNA levels 4 fold (Supplementary Figure S3C; Supplementary Table S1) and resulted in animals that reached larval stages but were developmentally delayed, smaller in size and died prematurely at early pupae stages. These mutants displayed a curved pupal case morphology with shorter spiracles, and failure of abdominal air bubble displacement (Figures 2C, D). This phenotype is characteristic in mutants of sarcomeric proteins, such as large Titin protein (Brooks et al., 2020), or Z-disk Trim 32 thin protein (LaBeau-DiMenna et al., 2012; Domsch et al., 2013) which are unable to properly contract muscles during the larval to pupal transition.

To determine the role of *prto* in muscle function we measured the pupal case axial ratios (length/width), which is a good indicator of muscle contraction during larval to pupal transition. The mean axial ratio value in *Mef2>UAS-Dcr;GFP-RNAi* controls and parental isogenic controls were <3 in accordance to previously published data (Brooks et al., 2020) (Figure 2E). In contrast, *prto* knockdown (*Mef2>UAS-Dcr;prto-RNAi*) resulted in a significantly higher mean axial ratio with a value >3.5 (Figure 2E). Similar phenotypes were produced by driving *prto* depletion with a different muscle driver (24B>) or by using with another *prto-RNAi* construct (#2) (Figure 2E). The muscle over-expression of either full-length wild-type *prto* (*UAS-prto-FL*) or Nt-tagged *GFP-prto* (*UAS-GFP-prto*) constructs together with *UAS-Dcr;prto-RNAi* partially rescued the pupal axial ratio towards wild-type (Figure 2E) and the lethality pupal phase (Supplementary Figure S3E) even considering that these constructs are still targeted by RNAi. In contrast, co-expression of *UAS-GFP* in muscles to control for the Gal4 dilution failed to rescue the *UAS-Dcr;prto-RNAi* axial ratio (Figure 2E). qRT-PCR experiments show that *prto* mRNA expression levels were restored to wild-type levels in *Mef2>UAS-prto-FL;UAS-Dcr* larvae carcasses (Supplementary Figure S3D; Supplementary Table S1) and larval somatic muscles seemed normal (Supplementary Figure S3H). Finally, when over-expression of *UAS-prto-FL* (increase of expression 16 fold change, Supplementary Figure S3D) or *UAS-GFP-prto* was driven with *Mef2-Gal4* driver in a wild-type background the larvae displayed normal larval somatic muscles (Supplementary Figures S3I, J–J′), the pupae showed wild-type axial ratios (Figure 2E) and viable offspring with normal muscle function was produced (not shown). Altogether these results indicate that the observed phenotype is specific to the reduction in *prto* function.

Next, we assessed the functional *prto* requirement in muscle contraction by monitoring L3 larval locomotion in an agar arena for 10 min (Berni, 2015). The exploratory behaviour of either parental *Mef2>UAS-Dcr* or *prto-RNAi* control larvae showed that they can fully move across the agar plate (Figure 2F) at an average velocity of 2 cm/min (Figure 2H) whereas *Mef2>UAS-Dcr;prto-RNA*i larvae velocity was greatly reduced producing a limited exploration of the arena (Figures 2G, H).

Taken together these findings demonstrate a strong correlation between *prto* levels and muscle function. Strong reduction of *prto* expression provokes severe L3 larvae locomotion defects and muscle contraction impairments at pupariation (axial ratios) whilst a milder reduction affects adult motor response and motility. Thus, the *prto* gene has an essential role in the regulation of muscle function and required further investigation.

### *prto* knockdown provokes sarcomeric defects and loss of muscle integrity

**FIGURE 3:**
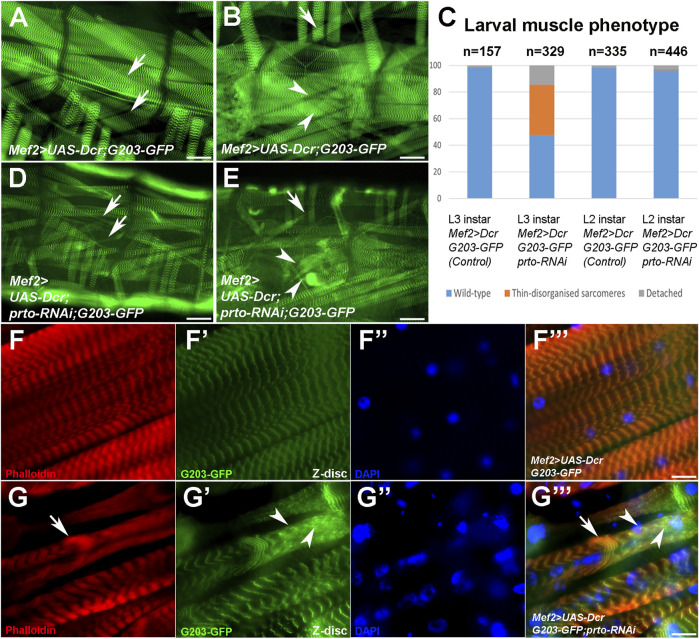
*prto* is required to maintain sarcomeric homeostasis. **(A–F)** Whole mount of L3 larvae muscle preparations expressing a GFP trapped Z-disc protein (203-GFP) in control **(A,B)** and *Mef2>UAS-Dcr;prtoRNAi* **(D,E)** larvae raised at 29°C and muscle phenotypic analysis of L2 and L3 somatic muscles **(C)**. Scale bar 50 µm. Arrows denote wild-type and over-stretched muscles and arrowheads wild-type and muscle detachments. Note in **(C)** ∼ 50% somatic muscles are thinned or truncated in *prto* deficient muscles in comparison to control. No phenotypic differences are observed in L2 instar. **(F–F‴)** Sarcomeric architecture of L3 control somatic muscle displaying actin filaments (phalloidin; **(F)**), Z-disks (203-GFP; **(F′)**), nuclei (DAPI; **(F′′)**) and merge **(F‴)**. Scale bar 20 µm. **(G–G‴)** Sarcomeric structure in *Mef2>UAS-Dcr;prto-RNAi* L3 muscles show a loss of the repeated sarcomeres with clumps of actin filaments (**(G)**, arrow), clusters of sarcomeric proteins distributed in the sarcolemma (**(G′)**, arrowheads) and nuclei being abnormal in shape and non-evenly distributed along the muscle **(G′′)** and merge **(G‴)**. Scale bar 20 µm.

To investigate the role of *prto* in larval musculature, we utilised a GFP-trapped Z-disc protein (*G203-GFP*, Crisp et al. (2008)) to monitor sarcomeric structures in control and *Mef2>UAS-Dcr,prto-RNAi* knockdown genetic backgrounds. Whole-mount larvae preparations of late L3 (100–120h AEL) showed a stereotypical array of 30 somatic muscles per hemisegment in controls (Figure 1B inset; Figures 3A–C (Bate, 1990)) whereas more than 50% of somatic muscles exhibited drastic morphological defects in *prto* KDs (Figures 3C–E). These defects were primarily classified as thinned muscles (38%) and detached muscles (14%) (Figure 3C). Interestingly, the somatic musculature of L2 *prto* KD larvae (65-70hr AEL) seemed normal and similar to the controls indicating that *prto* KD defects arise when the somatic muscles are rapidly growing and undergoing enormous mechanical stress (Figure 3C). Thus, Prto function seems not to be necessary for the formation of somatic musculature but instead is required for the maintenance of muscle integrity.

Next, we assessed the integrity of sarcomeric structures by monitoring the organisation of thin filaments (F-actin), and Z-disks (*G203-GFP*) in L3 larval fillets. Control larval somatic muscles showed regular repeated patterns of thin filament and Z discs along the myofibrils (Figures 3F–F‴; Supplementary Figures S3F–F‴). However, in *UAS-Dcr;prto-RNAi* KD muscles, myofibrils appear to be over-stretched and sometimes detached (Supplementary Figures S3G–G‴). In addition, the sarcomeric architecture is often disrupted by clumps of actin filaments (arrow; Figures 3H–H‴) and Z-disk proteins (arrowheads; Figures 3G–G‴). The *prto* KD muscles contained similar numbers of nuclei in comparison to controls (Supplementary Figures S4A–D) but they were disorganized and misshaped in comparison with control (Figures 3F″–FF‴, G″–G‴; Supplementary Figure S4). These *prto* KD muscle phenotypes are reminiscent to that of mutations of genes involved in sarcomerogenesis, such as *cofilin* (Balakrishnan et al., 2020), or *filamin* (*cherio (cher)* in *Drosophila*) (González-Morales et al., 2017; Brooks et al., 2020), which are necessary for the extensive larval muscle growth and the maintenance of muscle integrity respectively.

### Prto peptides show a punctate and striated pattern and are required to maintain proper endo-lysosome and autophagosome homeostasis in muscles

**FIGURE 4:**
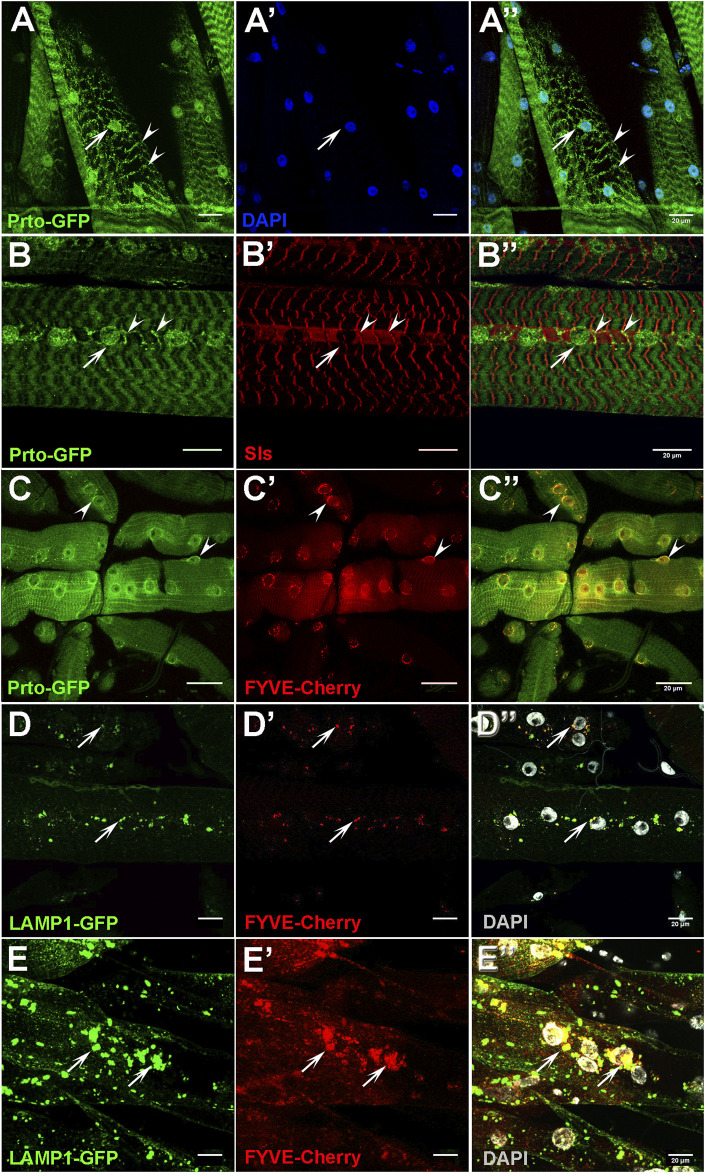
Prto localisation in endo-lysosomes and Z-disk compartments are important for muscle function. **(A–A′)** Expression of GFP-tagged Prto peptides in L3 somatic muscles shows localisation in the perinuclear region (arrow) and in stripes along the sarcolemma (arrowhead) **(A)**. Nuclei **(A′)**. Merge image **(A′′)**. **(B–B′)** Distribution of Prto-GFP **(B)** and the Sallimus (Sls) protein **(B′)** in L3 muscles. Prto-GFP is detected around the nuclei (arrow) and in a punctate pattern along the Z-disk (arrowheads) which colocalizes with Sls. Merged image **(B′′)**. **(C–C′)** Localisation of Prto-GFP **(C)** and FYVE-Cherry (endosome marker) **(C′)** in L3 somatic muscle. Note that co-localizations (arrowheads) are observed in the perinuclear region **(C′′)**. **(D–D′′)** Distribution of the lysosomal (Lamp1-GFP; **(D)** and the endosomal (FYVE-Cherry; **(D′)**) markers in the sarcolemma of control L3 somatic muscles. **(D′′)** Merged image with DAPI labelling nuclei (grey). Arrows denote co-localization near nuclei. **(E–E′′)** Endosome and lysosome distribution in *prto* KD L3 somatic muscles labelled as in **(D–D′′)**, showing an increase of endo-lysosomal aggregates in sarcolemma (arrows). Scale bars 20 µm.

To further explore the role of *prto* in sarcomeric homeostasis, we expressed a *GFP-tagged prto* construct in larval muscles, as it is partially able to fulfil the *prto* function *in vivo* (Figure 2E). Prto peptides showed a punctate and granulated pattern running along sarcomeres (arrowheads; Figures 4A–A″) and in perinuclear regions (arrow; Figures 4A–A″). Specifically, the Prto striated pattern was aligned to the Z-disks as it colocalise with Sallimus (Sls) proteins, which are large proteins that bind to myosin thick filaments within the Z-disks. Sls contribute to sarcomere extension properties of the muscle and are essential to maintain sarcomeric homeostasis and therefore may implicate Prto in muscle maintenance (Poovathumkadavil and Jagla, 2020) (arrowheads; Figures 4B–B″).

Next, we utilize different markers to precisely map Prto-positive cellular compartments. Prto peptides showed some degree of co-localisation with the endosomal FYVE-Cherry and lysosomal mRFP-spinster markers with the former enriched in the perinuclear regions (arrowheads; Figures 4C–C″) and the later in the sarcolemma (arrow; Supplementary Figures S6A–A″). This subcellular localisation is consistent with our earlier observations in embryonic S2 cells (Supplementary Figure S2F), suggesting a possible general *prto* role in these organelles. Importantly, these Prto-positive organelles are essential for maintaining muscle homeostasis and integrity as they are involved in cellular proteostasis of damaged sarcomeric proteins and tissue remodelling (Johnson et al., 2015; Fujita et al., 2017).

To understand the mechanisms by which Prto membrane peptides regulate the maintenance of sarcomeric structures we explored important cellular processes such as endosome and lysosome distribution. Firstly, in control muscles, the endosomes (FYVE-Cherry) and lysosomes (LAMP1-GFP) are distributed across the sarcolemma and enriched in the perinuclear region, overlapping abundantly in endo-lysosomal compartments (arrows; Figures 4D–D″). In *UAS-Dcr;prto-RNAi* KD muscles, these compartments are enlarged and accumulated around the aberrant nuclei, even when muscles are not showing major structural defects (arrows; Figures 4E–E″). Thus, it seems that disrupting *prto* function negatively affects endosome and lysosome functions and therefore compromises sarcomeric integrity leading to loss of muscle function.

### *prto* strongly interacts with the CASA pathway to regulate sarcomeric integrity

**FIGURE 5:**
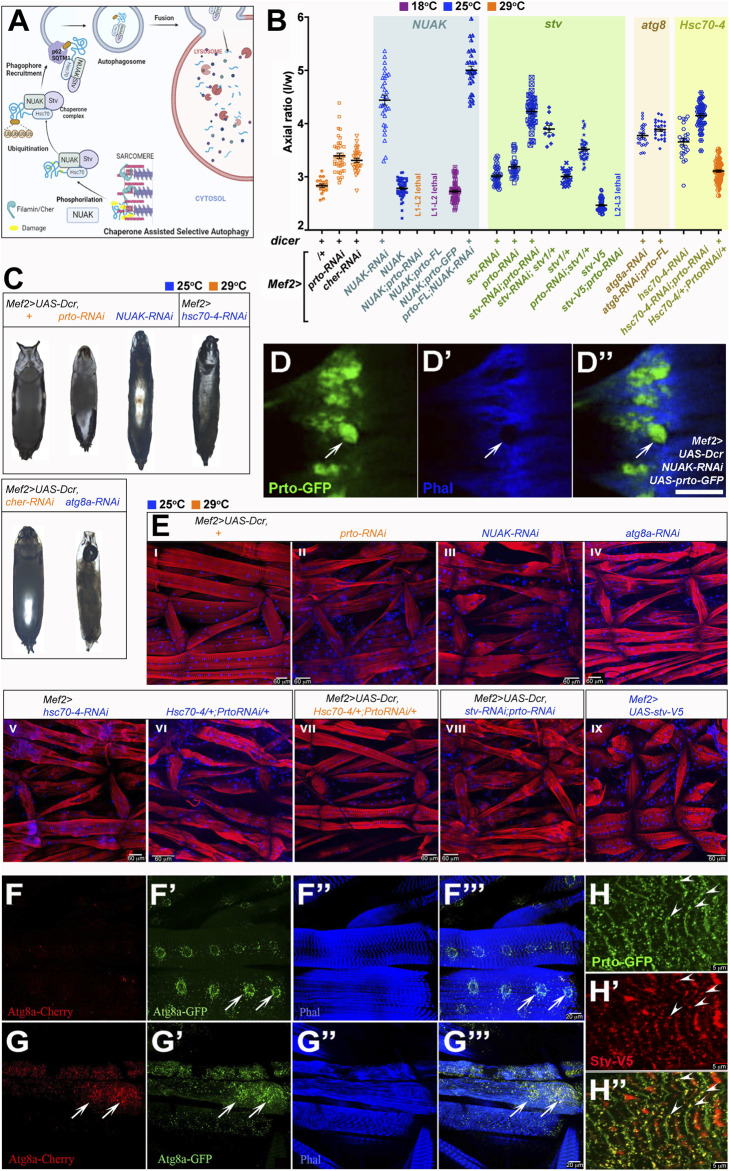
*prto* interacts genetically with CASA pathway for maintaining sarcomeric integrity. **(A)** Model for Chaperone Assisted Selective Autophagy (CASA) of Filamin/Cher in muscles constructed using Biorender.com. Filamin dimers maintain muscle sarcomeric structure undergoing sustained contractile forces during locomotion. Damaged Filamins (yellow) are phosphorylated by NUAK kinases and recruits Stv-Hsc70-4 protein complex for refolding. Unrepairably damaged Filamins are ubiquitinated and subsequently recruited by the p62/SQSTM1 complex and Atg8a to initiate autophagosome formation. Autophagosomes fuse with lysosomes for degradation of damage Filamin/CASA complexes. **(B)** Axial ratio measurements of *prto* and CASA RNAi KD genotypes and genetic interactions. Genotypes denoted in the x-axis and grouped by gene interaction each with a distinct background colour. All + and RNAi genotypes are driven with *Mef2>* without or with Dicer the latter denoted as (+) above genotypes. Temperature conditions for each genotype are shown as different colours (18°C: purple; 25°C: blue; 29°C: orange). 1way ANOVA with Bonferroni’s Multiple Comparison test. was utilised (see Supplementary Table S2). *n* = 11–63, error bars represent 95% CI. **(C)** Pupae morphology of following genotypes: *Mef2>UAS-Dcr* control, *Mef2>UAS-Dcr* driven: *prto-RNAi, NUAK-RNAi, cher-RNAi, atg8a-RNAi*, and *Mef2>hsc-70-4-RNAi*. Scale Bar is 1 mm. **(D–D′′)** Confocal image *Mef2>UAS-Dcr;NUAK-RNAi* muscle showing the expression of Prto-GFP **(D)** and actin filaments (Phalloidin) **(D′)**. Note that large aggregates of Prto-GFP are formed in actin-depleted regions in the sarcomeres (arrow). Merged image **(D′′)**. Scale bar 5 µm. **(E)** Sarcomeric structure and nuclei revealed by phalloidin (red) and DAPI (blue) staining respectively in L3 somatic muscles of stated genotypes*.* Scale bar 60 µm. **(F–F‴)** Autophagosome distribution revealed by expression of Atg8a-GFP-Cherry in L3 muscles shows increase of staining surrounding the nuclei (arrows). Autophagosomes expressing Atg8a-Cherry **(F)**. Expression of Agt8a-GFP only in neutral pH autophagosomes (not fused to lysosomes; **(F′)**). Actin filaments (phalloidin; **(F′′)**). Merged image **(F‴)**. **(G–G‴)** *Mef2>UAS-Dcr;prto-RNAi* L3 somatic muscle stained as **(F–F‴)**. Note a striking increase of autophagosomes is observed in the sarcolemma (arrows; compare with **(F)**. **(H–H′)** Image depicting Prto-GFP peptides (**(H)**; green), and V5-tagged-Stv protein (**(H′)**; red) showing colocalization (arrowheads) in punctae along Z-disks (**(H′)**; yellow). Scale bar 5 µm.

The Chaperone-assisted selective autophagy (CASA) pathway is a mechanism involved in the detection of misfolded sarcomeric proteins caused by contractile forces and their subsequent repair or their degradation *via* autophagocytosis (Arndt et al., 2010). Members of this pathway (Figure 5A) include: 1) the BAG3 (Starvin (Stv) in *Drosophila*)/Hsc70-4 Chaperone complexes that recognize defective proteins, such as Filamin which is named Cheerio (Cher) in *Drosophila*; 2) the NUAK kinases that phosphorylate these misfolded substrates for subsequent ubiquitination; 3) The p62/SQSTM1 complex that later binds ubiquitin chains of targeted proteins and triggers autophagosome formation and subsequent lysosomal degradation. Importantly, the functions of the members of the CASA pathway are conserved in metazoans (Arndt et al., 2010), and specific mutations in human homologues have been identified in Charcot-Marie Tooth type 2L, myopathy, cardiomyopathy, neuropathy and their over-expression is associated with poor prognosis in various cancers and also chemoresistance (Leber et al., 2016; Matsushima-Nishiwaki et al., 2017; Shen et al., 2018; Verdonschot et al., 2020; Cristofani et al., 2021; Kirk et al., 2021; Yerabandi et al., 2022). Notably, mutations in members of this pathway in *Drosophila* provoke muscle defects reminiscent of human myopathies. These include progressive accumulation of aberrant sarcomeric proteins, autophagosomal and lysosomal dysfunction leading to a progressive loss of muscle sarcomeric structures and contractile function (Arndt et al., 2010; González-Morales et al., 2017; Ader et al., 2022).

Since the *prto-RNAi* KD muscle phenotype (Figures 2, 3) shows a striking resemblance to those observed in CASA mutants we investigated possible genetic interactions between *prto* and members of CASA pathway. Our phenotypic characterisation showed that similar *prto*-like locomotive defects in adult flies and early death were observed in *Mef2*>*NUAK-RNAi* and in a lesser extent in *Mef2*>*cher-RNAi KD* males (Supplementary Figures S5A, B). Second, analysis of the Indirect Flight Muscle structure showed that *NUAK-RNAi* KD myofibrils were disorganized, and F-actin filaments were slightly faded mimicking the *prto-RNAi* phenotype (Supplementary Figures S5C–E). Third, strong reduction of gene functions of CASA members in muscles either by co-expressing *Dicer* or increasing temperature led to *prto-like* pupal abnormalities such as reduced/lack of anterior spiracles, air bubble retention and longer pupal case axial ratios (Figures 5B, C; Supplementary Table S2). These phenotypes correlated with the progressive deterioration of muscle sarcomeric structure (Figure 5E; Supplementary Figures S6D–D″) and accumulation of endo-lysosomal aggregates in the sarcolemma as observed in *prto-RNAi;UAS-Dcr* KD muscles (Supplementary Figures S6B-B″, C–C′; compare Figures 4E–E″). Based on these phenotypical similarities we wondered whether the increase of autophagic particles in muscles induced by CASA member mutations (Brooks et al., 2020) was also observed in *prto-RNAi;UAS-Dcr* KD muscles. Expression of a double fluorescent tagged Atg8a (autophagosome marker) in the muscle showed that *prto* KD muscles exhibit a remarkable increase in the number Atg8a positive puncta in comparison to controls (Figures 5F–F‴, G–G‴). Thus, these phenocopies at the cellular, physiological and morphological levels strongly suggest that *prto* function is closely associated to CASA-mediated protein turnover.

To further explore functional relationship between *prto* and the CASA pathway in sarcomeric homeostasis, we first tested the interaction with NUAK by monitoring the GFP-Prto peptide distribution in *NUAK-RNAi* KD muscles. Large Prto-positive aggregates were observed in actin-depleted areas in the sarcolemma in *NUAK-RNAi* KD mutants as previously reported with other Z-enriched sarcomeric proteins, such as Filamin and dCRYAB (Figures 5D–D′; Supplementary Figures S6D′–D″) (Brooks et al., 2020). We subsequently sought to assess the epistatic relationships among *NUAK* and *prto*. Decreasing *NUAK* function with *Mef2>NUAK-RNAi;UAS-Dcr* resulted in pupae that displayed the highest axial ratios and stronger L3 larval muscle abnormalities of the CASA members (Figures 5B, E–III). In addition, the over-expression of full length *UAS*-*prto* in *NUAK-RNAi* KD background, was unable to recover this phenotype and even enhanced the pupal case phenotype significantly (Figure 5B). A phenotype that also correlates with the formation of large Prto-positive aggregates as revealed by Prto-GFP in *NUAK* KD muscles (Figures 5D–D″). This suggest that *NUAK* and *prto* do not perform equivalent roles in muscles. Finally, over-expression of *NUAK* (*UAS-NUAK*) in muscles in a wild-type background results in pupal lethality, with pupae displaying slightly shorter axial ratios compare to controls (Figure 5B). Notably, either reduction or over-expression of *prto* gene function enhanced the *UAS-NUAK* phenotype (Figure 5B), with larvae that die in second or third instars before puparation. The over-expression of *prto-GFP* was less penetrant and did not change the *NUAK* gain of function phenotype. Thus, these results suggest that *prto* function could be acting downstream or in parallel to *NUAK* to modulate its function.

To address this hypothesis, we sought for possible genetic interactions between *prto* and the downstream members of the CASA pathway. Stv/BAG3 is able to bind NUAK, Cher, and the chaperone Hsc70-4 to form a complex that allows the ubiquitination of misfolded Cher proteins triggering their removal by autophagy (Arndt et al., 2010; Brooks et al., 2020) (Figure 5A). By using a specific RNAi construct and temperature conditions we sought a sensitive genetic background to underpin possible interactions. Decreasing *stv* (*stv-RNAi*) or *prto (prto-RNAi)* in muscles with *Mef2>UAS-Dcr* at 25°C produce offspring that reached adulthood and their pupal axial ratio were only slightly higher than controls (Figure 5B). Simultaneous knockdown of *stv* and *prto* resulted in pupal lethality and displayed a synergistic effect when we assessed the pupal axial ratio compared to each single gene knockdown including the strongest *UAS-Dcr*; *prto-RNAi* KD (29°C) pupae (Figure 5B), suggesting that both genes cooperate. This enhanced phenotype correlated with an increase in sarcomeric disruptions observed in the L3 muscles (Figures 5E–VIII compare with Figures 5E–II). Furthermore, removal of a single *stv* gene copy (*stv* ^*1*^ */+*) in a *prto-RNAi* KD background was also sufficient to further increase the *prto* KD pupal ratio (Figure 5B). Finally, the over-expression of *stv (UAS-stv-V5)* in muscles in a wild-type background produced short larvae (Tubby-like) with muscles shorter in length (Figures 5E–IX). The *Mef2>UAS-stv* offspring die at puparation and display reduced axial ratios compared to wild-type (Figure 5B). Importantly, *Mef2>UAS-stv* over-expression in *prto-RNAi* KD background was larval lethal at L2-L3 stage (Figure 5B). Altogether these results indicate that there is a strong genetic interaction between *ptro* and *stv* in maintaining sarcomeric homeostasis.

We next observed a genetic interaction between *prto* and the chaperone *hsc70-4*. The pupal axial ratio and sarcomeric larval muscle abnormalities of *prto-RNAi* and *hsc70-4-RNAi* double knockdowns were higher than each single gene knockdown (Figure 5B, compare E-VI with E-II and E-V), further supporting the role of Prto as a regulator of the chaperone complex required for protein assembly and turnover in muscles. Importantly, over-expression of *UAS-hsc70-4* was able to partially rescue the *UAS-Dcr;prto-RNAi* axial ratio phenotype at 29°C, suggesting that more efficient reassembling of unfolded sarcomeric proteins overcomes *prto* KD muscle abnormalities (Figures 5B, E–VII).

Finally, *atg8a* function acts at the base of the CASA pathway permitting the anchoring of the p62/SQSTM1, ubiquitinated cargo and chaperone complex and formation of autophagosome (Brooks et al., 2020). Reduction of *atg8a* function increases the pupal axial ratios and muscle sarcomeric abnormalities (Figures 5B, C, E–IV). Importantly, over-expression of *UAS-prto-FL* cannot rescue the *UAS-Dcr;atg8a-RNAi* knockdown phenotype (Figure 5B) suggesting that *atg8a* function is the limiting factor and that *prto* function could be acting on upstream members of CASA pathway. Altogether, the genetic interactions place Prto between NUAK and Atg8a functions and in close cooperation with the Stv/Hsc70-4 chaperone complex.

To ascertain how Prto peptides interact with CASA-protein turn over machinery, we monitored cellular localisation of GFP-Prto with Stv-V5 in muscles. Co-localisations of both factors were observed in punctae near the Z-disk region where active remodelling and turnover of Cher takes place (Arndt et al., 2010; Brooks et al., 2020) (arrowheads; Figures 5H–H′; Supplementary Figures S6E–E″). Supporting this view, FLAG-Prto peptides colocalised with the GFP-tagged short Cher isoform (Cher90) in the Z-disks (arrowheads; Supplementary Figures S6F–F″). Interestingly, a search of functional motifs (in diverse members of Prto peptide family) using ELM (Kumar et al., 2020) found an Atg8/LC3 binding domain in the Ct region of Prto peptides supporting their association to autophagosomes (Supplementary Figure S2C). Therefore, our genetic and cellular phenotypic characterisations indicate that Prto transmembrane peptides are involved in maintaining sarcomeric homeostasis, possibly by bridging the Stv/Hsc70-4 chaperone complex turnover machinery with membrane organelles such autophagosomes, and endo-lysosomes for degradation of misfolded Cher proteins. Further work would be required to identify if Prto directly interacts with any members of the CASA pathway.

### *prto* interacts with the CASA pathway to control growth in the wing discs

Refolding and degradation of damaged cargo proteins by the CASA pathway is important to maintain the cellular homeostasis in a diversity of developmental contexts, including muscles, neurons and kidneys (Höhfeld et al., 2021). Therefore, we wondered whether the regulation of CASA pathway by Prto peptides is a tissue-specific function or a broader one extending to other organs.

**FIGURE 6:**
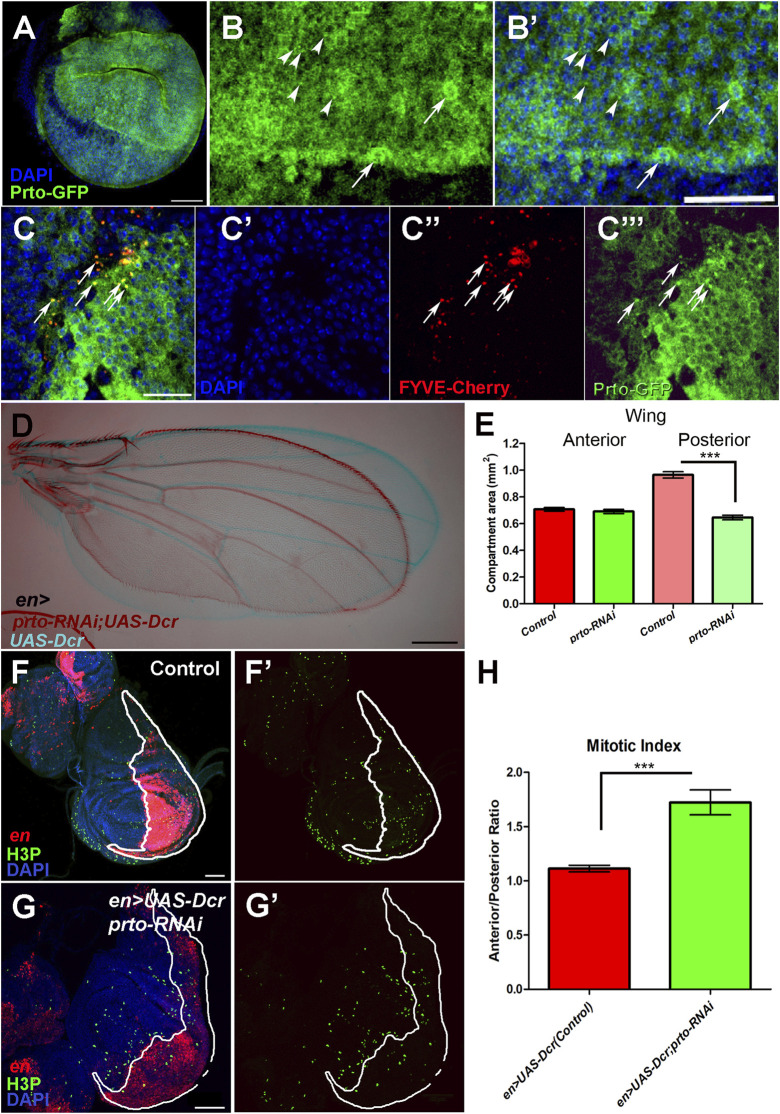
*prto* is required for proper growth of the *Drosophila* wing. **(A)** Prto-GFP expression in L3 imaginal disc using the *Dlmo-Gal4* driver shows that Prto peptides are localised in the plasma membrane. Scale bar 60 µm. **(B–B′)** Higher magnification of **(A)**, showing Prto-GFP peptides **(B–B′)** in the plasma membrane of epithelial cells and in intracellular puncta (arrowheads) and nuclei (DAPI, **(B′)**). Scale bar 40 µm. **(C–C‴)** Colocalization of Prto-GFP and FYVE-Cherry is observed in wing imaginal disc (arrows). Merged image **(C)** showing nuclei (DAPI; **(C′)**), endosomal marker (FYVE-Cherry **(C′′)**) and Prto-GFP **(C‴)**. Scale bar 20 µm. **(D)** Overlay of a control (*en>UAS-Dcr/+*; blue) and *en>UAS-Dcr;prto-RNAi* (maroon) adult wing showing a reduction of posterior region. Scale bar 200 µm. **(E)** Measurement of the anterior and posterior compartments of control and *prto* KD wings showing that the posterior compartment is reduced in *prto* KDs. Two-tailed Mann Whitney test was performed. *n* = 12, error bars represent 95% CI, *** = *p*< 0.0001. No significance *p* > 0.05 was observed in anterior compartment. (**(F–F)**′ Distribution of Histone 3 phosphorylation (green) in control *en>UAS-dsRed* wing imaginal discs and posterior compartment labelled in red. **(G–G′)** *en>UAS-dsRed;prto-RNAi* wing imaginal disc labelled as in **(F)**. Scale bar 50 µm. **(H)** Mitotic index ratio is the number of positive H3P cells anterior compartment divided by the number positive H3P in posterior compartment (white outlined red staining). Control wing imaginal discs is around 1. However, the mitotic index ratio in *en>UAS-dsRed;prto-RNAi* is higher due to a lower number of cells in posterior compartment undergoing mitosis. Two-tailed Mann Whitney test was applied. *n* = 13, error bars represent 95% CI, *** = *p*< 0.0001.

We explored the role of *prto* and the CASA pathway in wing development since *prto* is highly expressed in wing imaginal discs and there are no insights into CASA-mediated functions in this proliferative epithelial organ. First, we expressed a *GFP-tagged prto* construct with the *Dlmo-Gal4* driver in the wing discs (Figures 6A, B–B′) and found that GFP-Prto peptides were localised in the plasma membrane (arrows) and in intracellular punctae in epithelial cells (arrowheads). Some of these Prto-positive intracellular compartments were labelled with the FYVE endosomal marker (arrows; Figures 6C–C‴), suggesting that Prto subcellular distribution across different cell types seems to be conserved.

To investigate the role of *prto* in wing development we knocked down *prto* expression in different regions of the wing imaginal disc by expressing the *prto-RNAi* construct with different Gal4 drivers (*69B*, *Dlmo*, *engrailed (en)*) at different temperature conditions. A specific and significant reduction of the size of the wing was observed in the induced *prto-RNAi* region (Figures 6D, E; Supplementary Figure S7). For example, *prto-RNAi* knockdown in the posterior compartment causes shortening of the posterior region of the wing without affecting the anterior part (Figures 6D, E). By counting the number of wing cells (trichomes) in the anterior and the posterior regions of the adult wings we determined a significant ∼30% reduction in number of cells in *en>UAS-Drc;prto-RNAi* wings in comparison to *en>UAS-Dcr* controls (Supplementary Figures S8A, B). Similarly, reduction of *prto* function with *Dlmo-Gal4*, a driver strongly expressed in the dorsal compartment, produces a curled wing phenotype (not shown) due to reduction of the dorsal epithelium (Supplementary Figure S7).

To ascertain which cellular processes lead to a reduction of wing epithelial cells in *prto-RNAi* KDs we first investigated the effect of *prto* function on apoptosis and proliferation in the developing wing imaginal discs. Reduction of *prto* using *en>* driver produced posterior compartments of an abnormal shape (misfolded epithelium) with over-stretched epithelial cells showing larger gaps among neighbouring cells. Despite this phenotype, no major increase on apoptosis was observed in *prto-RNAi KD* wing discs (Supplementary Figures S8C, D–D‴, F–F‴). By contrast, a significant decrease in mitosis was observed by comparing the number of phosphorylated Histone 3 (H3P) positive cells in the anterior (control) and posterior (KD) wing compartments (Figures 6F–F′, G–G′). The Mitotic index between Anterior/Posterior compartment was ∼30% higher in *prto-RNAi* KD than in controls (Figure 6H), suggesting a reduction in the number of mitotic cells following *prto* depletion which correlates with the reduction in cell number observed in *prto-KD* adult wing compartment (Supplementary Figures S8A, B).

**FIGURE 7:**
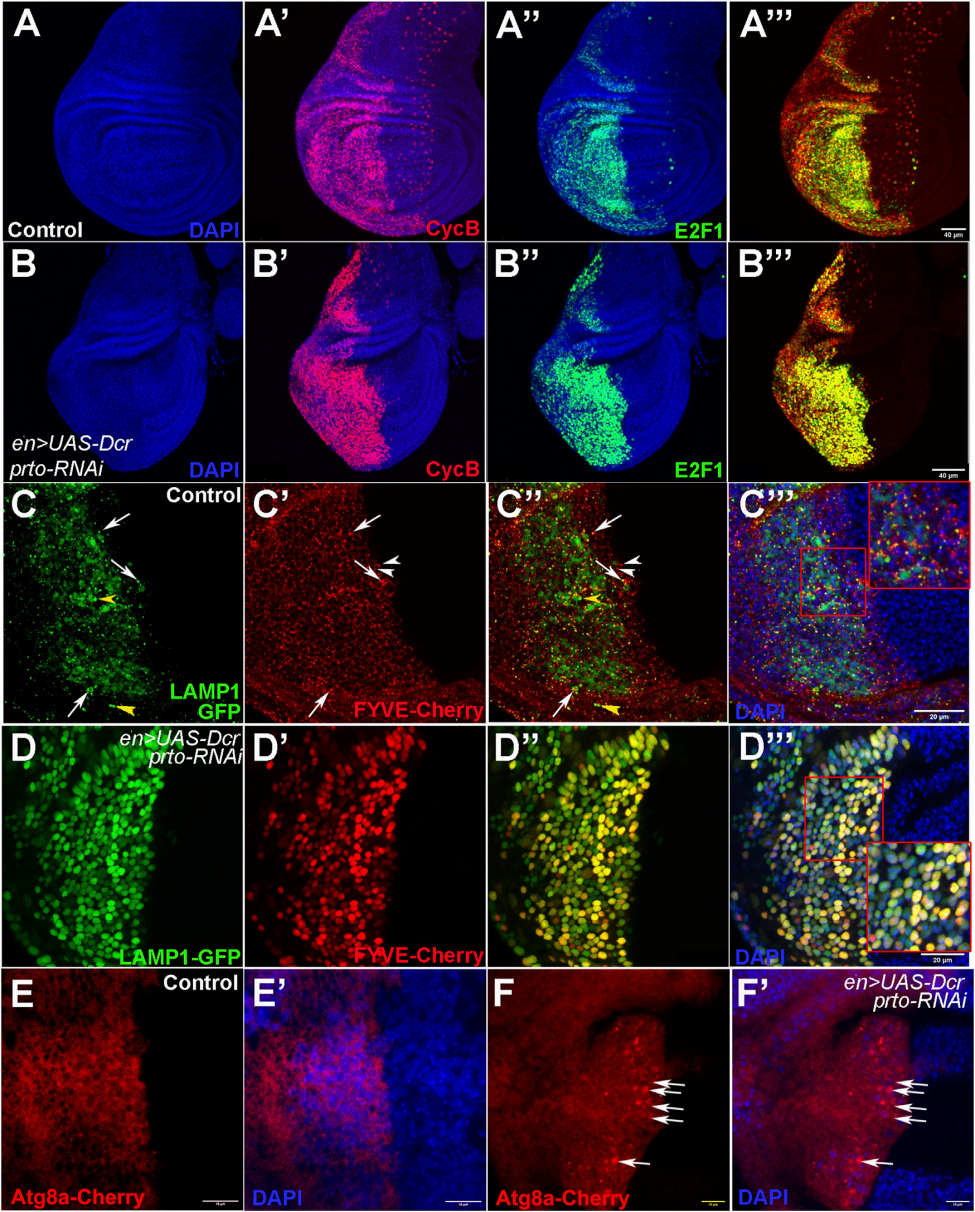
Cell cycle progression and endo-lysosomal trafficking is disrupted in *prto* KD epithelial wing cells. **(A–A‴)** Expression of the FUCCI system in the posterior compartment using *en>*driver. **(A)** DAPI expression labelling the nuclei. **(A′)** DAPI and NLS-dCycB-mRNP expression labelling cells in S and G2 phases and entering Mitosis. **(A′)** DAPI and dE2F1-GFP expression labelling cells in G1, G2 and mitosis. **(A‴)** NLS-dCycB-mRNP and dE2F1-GFP expression. **(B–B‴)** FUCCI expression in the posterior compartment in *prto* KD wing imaginal disc labelled as in **(A)**. Note that more epithelial cells in *prto* KD wing discs seem to be expressing both cell cycle markers suggesting that cells could be a stalled G2-Mitosis transition. Scale bar 40 µm. **(C–C‴)** Expression of lysosomal (LAMP1-GFP; **(C)**) and endosomal (FYVE-cherry; **(C′)**) markers in the posterior compartment in control wing imaginal discs. Merged image (C″) and with nuclei (DAPI; **(C‴)**). A distinctive puncta pattern of endosomes (white arrowheads) and lysosomes (yellow arrowheads) is observed in the cytoplasm of epithelial cells. Co-localization of both markers are highlighted with an arrow. Higher magnification is shown in inset in **(C‴)**. Scale bar 20 µm. **(D–D‴)** *prto* KD wing imaginal disc labelled as in **(C)**. The patterns of endosomes and lysosomes have become broader. Higher magnification is shown in inset. Scale bar 20 µm. **(E,F)** Expression of an *Atg8a-Cherry* construct in the posterior wing compartment with *en>UAS-Dcr* control **(E)** and with *prto-RNAi* **(F)**. Merge with DAPI **(E′)** and **(F′)** respectively. An increase of aberrant autophagosomes in posterior is observed in *prto* KD discs (arrows). Scale bar 20 µm.

To further explore the role of *prto* in growth we monitored the cell cycle stages of wing epithelial cells using the FUCCI system. The FUCCI system compares expression of two different cell cycle stage protein domains tagged to different fluorescent proteins simultaneously (E2F1-GFP and CycB-RFP) allowing the identification of cell cycle phase by fluorescence (Zielke et al., 2014). Most of wing epithelial cells divide in a non-synchronic fashion apart from those in the wing margin (Barrio and Milán, 2020). In control wing imaginal discs (*en>UAS-Dcr*), FUCCI expression in the posterior compartment revealed a mosaic of cells expressing specific fluorescent cell-cycle markers showing cells at different stages of the cell cycle (Figures 7A–A‴). In contrast, the *UAS-Dcr;prto-RNAi KD* wing discs showed a qualitative enrichment of cells co-expressing both cell cycle markers (yellow) indicating that cells were stalled in G2 phase (Figures 7B–B‴). We next performed relative quantifications of numbers of cells expressing each cell cycle markers by the automatic image processing DeadEasy Mito-Glia method (Forero et al., 2010). A similar and steady increase in the population of cells expressing each of the cell cycle markers was observed in *en>UAS-Dcr,prto-RNAi* in comparison to controls (Supplementary Figure S8E). Since *prto-RNAi* KD wing regions show lower number of cells going through mitosis, this relatively equal increase in both of these cell cycle populations in *prto-RNAi* KDs suggests that they are arrested at G2/M transition. Altogether, these findings show that *prto* function is important for proper cell proliferation in the wing imaginal epithelium.

Next, we monitored the distribution of endosomes and lysosomes (Prto-positive intracellular compartments) in the wing disc epithelium by using FYVE and LAMP-1 markers respectively. Both the FYVE and LAMP-1 markers are distributed in cytoplasmic punta in *en>UAS-Dcr* control wing imaginal discs (Figures 7C–C‴). However, in *en>UAS-Dcr; prto-RNAi* KD wing discs, larger aggregates of FYVE and LAMP-1 were observed throughout the cell (Figures 7D–D‴). Interestingly, an increase of Atg8a-positive autophagosomes was also observed in *prto-RNAi KD* wing discs in comparison to controls (Figures 7E–E′, F–F′). Thus, these results suggest that *prto* depletion disrupts endo-lysosomal homeostasis and provokes an accumulation of autophagosomes leading to a decrease of cell proliferation in wing disc epithelium.

**FIGURE 8:**
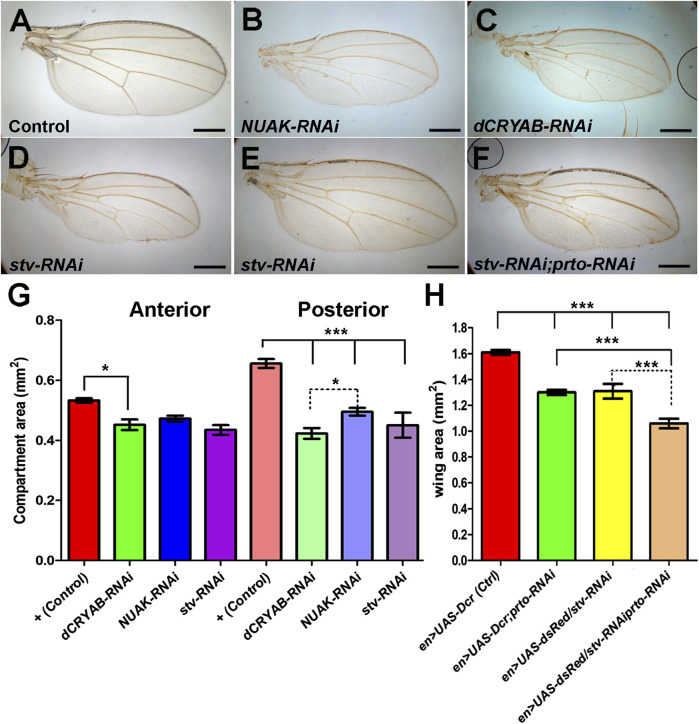
CASA members interact with *prto* for proper wing growth. Adult wings of Control **(A)**, *NUAK-RNAi* **(B)**, *dCRYAB-RNAi* **(C)** and *stv-RNAi* **(D)** knockdown male flies using *en>UAS-dsRed* driver. **(E,F)** Adult female wing of a single *stv*-RNAi knockdown **(E)** and double *prto-RNAi* and *stv-RNAi* knockdown **(F)** using *en>UAS-dsRed* driver. **(G)** Area of anterior and posterior compartments of adult male wings showing that knocking down CASA members, *NUAK*, *dCRYAB*, *stv*, using *en>UAS-dsRed* driver produces a reduction in size of the posterior compartment. 1way ANOVA with Bonferroni post test. *n* = 24–29, error bars represent 95% CI *** = *p*< 0.0001, ** = *p*< 0.001, * = *p*< 0.05. **(H)** Area measurements of entire female adult wings showing similar reductions of wing size provoked by single *prto*, and *stv* RNAi knockdowns. Further wing size reduction is achieved in the double knockdowns. 1way ANOVA column statistics with Bonferroni post test. *n* = 10–39, error bars represent 95% CI *** = *p*< 0.0001.

Since *prto* knockdowns alter endo-lysosome and autophagosome pathways in both somatic muscles and wing imaginal discs, we investigated whether Prto-mediated regulation of the CASA pathway is required for proper wing growth. Knocking down either *NUAK* or *dCRYAB* by RNAi in the posterior compartment using the *en-Gal4* driver provoked a reduction in the posterior wing area in comparison to the control anterior wing area (Figures 8A–C, G). In addition, the *stv-RNAi* knockdown using e*n-Gal4* driver resulted in extensive male pupal lethality, although some adult escapers eclosed displaying wings with the posterior compartment reduced (Figures 8D, G). Thus, these wing size reductions in KD of CASA members phenocopy those of *prto-RNAi* KD flies.

Finally, we tested whether genetic interactions between *prto* and CASA members exist in the wing. First, *en>stv-RNAi* knocking down produced females that reached adulthood displaying reduced wings (Figures 8E, H). Reduction of *prto* function in *stv-RNAi* KD females enhanced all wing phenotypes resulting from the independent knockdown of each genes (Figures 8F, H). Similarly, removing a single functional *stv* gene copy (*stv* ^*1*^ */+*) in a *UAS-Dcr;prto-RNAi* background further reduced the posterior compartment area with minimal anterior compartment defects (Supplementary Figures S8G–J). Therefore, the observed genetic interactions between *prto* and genes in the CASA pathway resemble those found in muscles and suggest a putative role of Prto peptides and the CASA pathway in controlling protein turnover to maintain correct growth of the wing imaginal disc.

## Discussion

In this work, we have functionally characterised *purriato*, a member of a new short CDS gene family which is essential for maintaining sarcomeric integrity and proper growth in *Drosophila* somatic muscles and wing imaginal discs respectively. Our BLAST and hmmer searches (Johnson et al., 2008; Finn et al., 2011) have found Prto homologues in species throughout all major taxonomic groups within Protostomia (Arthropoda, Nematoda, Annelida, Mollusca) indicating these Prto peptides belong to an ancient family. Based on Prto conservation, the most plausible explanation is that the Prto gene arose at least >650 million years ago when Protostomes and Deuterostomes diverged. However, since the methods for identification of homologies (BLAST) are not well suited for smORFs, it is possible that Prto homologues might exists in Deuterostomia but their accumulated divergence does not allow their identification due to the limitations of pairwise comparisons for small ORFs.

The secondary structure analysis of the Prto family has shown that these are single transmembrane peptides supporting the previous findings that these domains are enriched in the short CDS smORF class (Aspden et al., 2014; Couso and Patraquim, 2017). Using ELMI, we have also identified a conserved PP2 phosphatase binding site and Atg8/LC3 interacting domain in the N-terminal and C-terminal regions respectively. In addition, expression of a tagged-Prto constructs in different tissues showed that Prto peptides are enriched in specific intracellular compartments, such as endosomes and lysosomes suggesting a common role for Prto in processes involving these organelles, as previously observed with other conserved shCDSs, such as Hemotin or SVIP (Pueyo et al., 2016b; Johnson et al., 2021).

ModEncode RNA-seq data have revealed that *prto* mRNA is broadly expressed at different life cycle stages and organs, but it is specially enriched in whole body (mainly muscles and epithelium), gut, and imaginal discs (Brown et al., 2014). We have corroborated *prto* expression in these tissues by *in situ* hybridisation. Interestingly, *prto* homologues are also expressed in some of these tissues, such as somatic muscles in other protostomes lineages suggesting they could be involved in regulating similar cellular and physiological processes.

Our phenotypic characterisation has shown that *prto* is an essential gene required for maintaining cellular homeostasis. RNAi-mediated knockdown of *prto* in muscles produce offspring with shorter lifespan showing climbing abnormalities, as previously reported (Schnorrer et al., 2010). Enhancing the reduction of *prto* function using *UAS-Dicer* overexpression in this tissue results in a progressive loss of sarcomeric integrity and disorganised myofibrils leading to striking reduction of the motility of L3 larvae, pupal case abnormalities and pupal lethality. Interestingly, *UAS-Dcr,prto-RNAi* KD larval muscles display large endo-lysosomal aggregates plus abnormal granulated and misshaped nuclei.

We have also revealed that *prto* is involved in regulating proliferation of epithelial cells in the wing imaginal disc. Knocking down *prto* gene function using specific Gal4 drivers provoked a reduction of the respective wing region. Our analysis has shown that there is a striking reduction in the number of trichomes (which mark the wing cells) which is likely driven by a reduction of mitotic cells in the developing *prto-RNAi* KD wing imaginal disc. Consistent with these results, the cell cycle marker patterns in *prto-RNAi* KD wing cells suggest that they could be stalling at G2 and failing to enter mitosis. Finally, the reduction of *prto* produces changes in the endosome and lysosome cellular distributions from a punctuate pattern to a broader one throughout the cell in *prto-RNAi* KD wing discs. This phenotype could be due to the stimulation of endosomal and lysosomal biogenesis in G2 cell cycle phase (Yin et al., 2020) but also concomitantly being caused by an increase of aberrant endo-lysosomes as previously observed in muscle cells. Finally, it is important to note that *prto-RNAi* KD in follicular cells produces epithelium ruptures, which is a phenotype associated to defects in growth as observed in cell cycle regulators mutants (Berns et al., 2014). Thus, the role of *prto* in regulation of growth is of wider relevance as it is extended to other organs.

This study has revealed a close genetic relationship between Prto and members of the CASA pathway. CASA is a conserved mechanism for protein turnover necessary to maintain cellular homeostasis. This pathway contains chaperones (HSP) and co-chaperones involved in the surveillance and refolding of mechanically damaged client proteins. When damaged proteins are beyond repair, BAG3/Stv recruits CHIP, a ubiquitinin-ligase, that adds ubiquitin to the target. Ubiquitinated cargo is then recognized by p62/ref(2)P and consequently recruits autophagophores. Finally, phagophores encircle the cargo becoming autophagosomes and then fuse to lysosomes for cargo degradation (Arndt et al., 2010). Reduction of expression of CASA members produce phenotypes that mimic those observed in *prto* depleted muscles and wings. These include lifespan shortening, adult climbing and larval motility defects both caused by loss of sarcomeric organisation of IFM and larval somatic muscles respectively, and reduction of wing size.

Our genetic analyses have shown that modulation of *prto* function is able to enhance the CASA mutant phenotypes. For example, simultaneous loss of *prto* and *stv* or *hsc70-4* functions increased the pupal axial ratio and severity of muscle sarcomeric disorganisation above that of when they are depleted alone. Furthermore, overexpression of *prto* also enhanced the *NUAK* loss of function axial ratio phenotype. Importantly, increasing the folding capability by overexpressing *UAS-hsc70-4* was sufficient for partially rescuing the *prto-RNAi* KD muscle phenotype. Crucially, genetic interactions in the wing resemble those in the muscles in that concomitant reduction of *prto* and *stv* functions enhances the reduced wing phenotype observed in each single gene KDs and *prto* overexpression causes pupal lethality in a *NUAK*-*RNAi* background. Altogether these findings suggest that the correct Prto levels are key for the regulation of CASA pathway for maintaining sarcomeric integrity and wing growth.

In muscles, GFP-tagged Prto peptides are localised in a punctate pattern, which is enriched in the perinuclear region and in the Z-discs; the latter is composed of a supramolecular scaffolding protein network involved in maintaining sarcomeric organisation. In the Z-discs Filamin (Cheerio) proteins form dimers that interact with actin filaments and integrins acting as a mechanosensory hinges which keep sarcomeric integrity while sarcomeres are undergoing mechanical stress. Filamin is one of the main cargoes for the CASA pathway and interacts with BAG3/Stv for refolding or degradation in muscle cells. This study has identified a novel regulator of the CASA pathway. Firstly, we have shown that GFP-tagged Prto peptides colocalise with Stv-V5 in Z-discs in larval muscles and secondly there are striking similarities in the molecular processes affected in CASA members and *prto* KDs, such as accumulation of autophagosomes, and aberrant endo-lysosomes and aggregation of sarcomeric proteins. Although further biochemical and molecular approaches are needed to understand the mechanisms of Prto modes of action, the identification of an Atg8/LC3 interacting domain in Prto transmembrane peptides and their frequent localisation in endosome and lysosome compartments suggests that they could be involved in the recruitment of CASA complexes into autophagosomes and/or subsequent maturation.

Our work has also revealed that *prto* could be an intrinsic regulator of the CASA pathway, as *prto-RNAi* KD muscles display phenotypes such as granulated and misshaped nuclei, which are not related to the cargo filamin function (Brooks et al., 2020). Importantly, it has been recently reported that the CASA pathway is involved in the age-dependent turnover of nuclear Lamins in IFM muscles (Ryan et al., 2021). This process requires the interaction between p38Kb/Stv and their loss of function produces abnormally shaped nuclei showing Lamins aggregates (Ryan et al., 2021). Therefore, one could hypothesise that Prto peptides could regulate Stv-mediated degradation of Lamins in muscles but also in wing imaginal discs to maintain nuclear and cytoskeletal organisation and genome stability (Dialynas et al., 2010; de Leeuw et al., 2018; Ryan et al., 2021). Thus, Prto seems to be an important factor for the regulation of CASA mediated turnover of a diversity of client proteins involved in maintaining sarcomeric and nuclear integrity in muscles and growth in wing discs. Future work exploring any direct interaction between Prto and CASA members would be important to further unlock this regulatory mechanism.

The CASA pathway is a conserved mechanism necessary to maintain cellular homeostasis under mechanical stress. Mutations of CASA members in humans lead to severe human diseases, including skeletal muscle myopathies, cardiomyopathies (Martin et al., 2021), kidney filtration failure (proteinuria) (Perico et al., 2016), Alzheimer disease and other neuropathies (Counts et al., 2014; Datta et al., 2017; Ji et al., 2019; Adriaenssens et al., 2020). Therefore, understanding the modes of action of Prto peptides in the regulation of CASA pathway would not only increase our knowledge on this important mechanism in controlling proteostasis but could also lead to the identification of new targets and development of potential agents to counteract the CASA members’ mutations. Thus, this study is another example showing the relevance of smORFs in regulating important and conserved cellular processes and stresses the importance of their annotation in the genomes (Waldron et al., 2015; Mudge et al., 2022), association with diseases and their systematic characterisation to uncover the full potential of the small ORFeome.

## Data Availability

The original contributions presented in this study are included in the article/Supplementary Materials, further inquiries can be directed to the corresponding author.

## Supplementary material

SUPPLEMENTARY FIGURE S1 Prto genomic locus, mRNA expression and translation. **(A)** Genomic locus of the *Purriato (prto)* gene (rectangle) as depicted in Genome Brower (Flybase). **(B)** IGV genome browser displaying the *prto* gene annotation and the open reading frames (red arrow; bottom), the mRNA-seq track reads (middle) and footprint (Ribo-seq) read track (top). **(C–F)** Embryonic *prto* mRNA pattern of expression revealed by *in situ* hybridisation Scale bar 50 µm. **(C)** Maternal *prto* mRNA contribution in stage 3 embryo. **(D)** Ventral view of a stage 6 embryo showing expression in the mesoderm layer (arrow) and in the cephalic furrow (arrowhead). **(E)** Stage 10 embryo starting germ band retraction showing expression in the mesoderm. **(F)** Dorsal view of a stage 14 embryo showing *prto* mRNA expression in the visceral mesoderm (arrows).

SUPPLEMENTARY FIGURE S2 Prto peptide secondary structure, conservation and subcellular localisation in S2 cells. **(A)** Prediction of alpha helices (red) and beta-sheet (green) secondary structures based on JNET prediction software. **(B)** MAFFT alignment of the Prto peptide sequences used in Figure 1 constructed by GUIDANCE 2 showing the good confidence scores with a Guidance alignment score of 0.897454 (n# alternative bootstrap guide-trees: 100). **(C)** Clustal W of members of Prto family representing members from main clades (Arthropoda; Nematoda; Anelida, Moluska) highlighting the presence of conserved domains such as transmembrane domain, PP2A docking domain and LIR domain, which binds Atg8. **(D–D′′)** C-terminus Venus tagged Prto **(D′)** and N-terminus Flag tagged Prto **(D′′)** expressions in *Drosophila* S2 cells showing different tagging approaches does not affect Prto subcellular localisation. Scale bar 5 µm. **(E)** Mander’s coefficients shows high levels of co-localization from different tagged Prto peptides. **(F–F′′)** Distribution of Prto-GFP and Hemo-FLAG peptides in *Drosophila* S2 cells. Scale bar 5 µm. **(G–G′)** Distribution of the Prto-GFP and Mitotracker in S2 cells. Scale bar 1 µm. **(H–H′)** Distribution of Prto-GFP and Lysotracker in S2 cells. Scale bar 5 µm. **(I)** Co-localisation measurements (Manders’s coefficient) showing that Prto peptides co-localize with the endosome Hemotin peptide. Colocalisation of Prto with other markers of subcellular organelles such as lysosomes or mitochondria is also found but a much lesser extent. Two tailed Mann Whitney test was used to compare each pair samples. *n* = 11–13, error bars represent 95% CI, *** = *p* < 0.0001.

SUPPLEMENTARY FIGURE S3
*prto-RNAi* knockdown IFM and larval somatic muscle phenotypes. **(A,B)** Myofibrils of indirect flight muscles (IFM) of a control **(A)** and of *prto-RNAi* KD adult flies **(B)**. Note that myofibrils seemed less compacted, and sarcomeres slightly faded in *prto-RNAi* KD. **(C)** qRT-PCR demonstrating the knockdown of *prto* in larvae carcasses of *Mef2>prto-RNAi* with or without *UAS-Dcr* at 25°C (Blue) or at 29°C (orange). *Mef2>UAS-Dcr/+,* and *prto-RNAi/+* lines were used as controls. n > 3, errors bars represent SD and statistical analysis is shown in Supplementary Table S1. Note that both higher temperature and the expression of Dicer maximize *prto* knockdown. **(D)** qRT-PCR showing the *prto* mRNA fold change in controls (red) and *prto* knockdowns (green) at 29°C from C and *prto* overexpression (UAS-prto) in a knockdown (blue) or wild-type (brown) backgrounds. n > 3, errors bars represent SD and statistical analysis is shown in Supplementary Table S1. Note the overexpression of *prto* rescues the *prto* mRNA expression in KD larvae and substantially increases the levels in a wild-type background. **(E)** Lethality test showing percentage of individuals dying at specific life cycle stages and showing distinctive phenotypes (i.e., curved pupae) in *Mef2>prto-RNAi;UAS-Dcr*; in a wild-type (+), *UAS-Prto* and *UAS-Prto-GFP* backgrounds. Note that there seems to be a shift in the lethality phases towards later stages in both *UAS-Prto* and *UAS-Prto-GFP* but with lesser extend in the latter. **(F)** Actin filaments (phalloidin staining). **(F′)** Z-disks (203-GFP). **(F′)** myonuclei (DAPI). **(F′)**. Merged image in **(F‴)**. Scale Bar 100 µm. **(G–G‴)** *UAS-Dcr;prto-RNAi* KD L3 fillets labelled as in **(F)**, showing somatic muscles over-stretched and thinned, Scale Bar 100 µm. **(H)** Somatic muscles labelled with phalloidin (red) and DAPI (blue) of a *Mef2>UAS-Dcr;prto-RNAi;UAS-Prto* L3 larvae raised at 29°C displaying a fairly normal muscle pattern Scale Bar 60 µm. **(I)** Somatic muscles labelled as H of a L3 larvae overexpressing *prto* full length transcript with Mef2 driver at 29°C. Note that muscles appear normal and these larvae pupate and hatch into viable adults. Scale Bar 60 μm **(J–J′)** Larval somatic muscles overexpressing *UAS-Prto-GFP* (green) at 29°C labelled with phalloidin (red) and DAPI (blue). **(J)** merge image showing punctated striped Prto-GFP pattern in muscles. **(J′)** Light sarcomeric filaments (red) and myonuclei (blue) appear normal.

SUPPLEMENTARY FIGURE S4
*prto* is necessary to maintain regular nuclei shape. **(A–C)** Somatic muscles of L3 larvae showing Actin filaments (phalloidin; Red) and myonuclei (DAPI, blue) in Control **(A–A′)** and *prto-RNAi* KDs **(B,C)**. Note that nuclei appear deformed and unevenly distributed in *prto-RNAi* KD in comparison with controls. Scale bar 60 µm. **(D)** Counting of number of nuclei per lateral transversal muscle (LT2, LT3 and LT4) shows that there not seem to be a consistent difference in the number of nuclei. Two-tailed t-test statistics were performed to compare nuclei number of controls vs. *prto-RNAi* for each muscle type. *n* = 3–8, error bars represent 95% CI, * = *p* < 0.05. **(E–E′′)** Nuclear envelop of nuclei revealed with Lamin antibody staining (red) in control L3 somatic muscles and phalloidin (green). Scale bar 60 µm. **(E′)** ImageJ binary image for nuclei detection. **(E′′)** ImageJ nuclei area measurements in L3 somatic muscles. **(F)** Nuclei area measurements show that *prto-RNAi* KD nuclei in general seemed not only larger but also display wider variation in size than controls. Two-tailed Mann Whitney test was carried out. *n* = 125–150, error bars represent 95% CI, *** = *p* < 0.0001.

SUPPLEMENTARY FIGURE S5 Knocking down functions of CASA members produce *prto-like* muscle phenotypes. **(A)** Averaged viability from 3 independent survival assays of Prto and CASA member knock down male adults showing that *NUAK*-RNAi mimics *prto-RNAi* effects on survival. *n* = 7–12 males. **(B)** Climbing assays showing that in general CASA mutants had their locomotion ability reduced, *prto* and *NUAK* KDs being the most affected. 1way ANOVA statistics was used to compare climbing among genotypes at the same time periods. *n* = 15 per replica, error bars represent 95% CI. **(C–E)** Myofibrils of IFM thoracic adult muscles in control **(C)**, *prto-RNAi* **(D)** and *NUAK-RNAi* **(E)** showing actin filaments (green) and nuclei (red). Disorganisation of myofibrils and faded sarcomeres are observed in *prto* and *NUAK* KDs.

FIGURE SUPPLEMENTARY S6 CASA members colocalise with Prto-GFP peptides and their knockdowns produce aberrant endo-lysosomal patterns in larval muscles. **(A–A′′)** Spatial distribution of Prto-GFP peptides (**(A)**, green) and the lysosomal marker, Spinster-mRFP (**(A′)**, red) in L3 somatic muscles. Numerous positive Prto- membrane vesicles and punctae are associated with lysosomes (arrow; **(A′′)**). Scale bar 20 µm. **(B–B′)** Distribution of lysosomes labelled with Lamp1-GFP (B, green), endosomes labelled with FYVE-cherry (**(B′)**, red) and merge image with nuclei labelled with DAPI **(B′′)** in *NUAK* KD L3 somatic muscles. Arrows denote clusters of accumulation of aberrant endo-lysosomes. **(C–C′)** Localisation of lysosomal **(C)** and endosomal **(C′)** organelles and merge image with nuclei **(C′′)** labelled as in B in *atg8a* KD L3 somatic muscles. Arrows denote clusters of accumulation of aberrant endo-lysosomes. **(D–D′)** Localisation of Prto-GFP peptides **(D)** and actin filaments **(D′)** in *Mef2>UAS-Dcr;NUAK-RNAi* L3 somatic muscles. Note that muscles are over-stretched and broken showing loss of sarcomeric architecture and aberrant clusters of Prto-GFP in sarcolemma (arrows). Merged image **(D′′)**. Scale bar 60 µm. **(E–E′′)** Lower magnification image shown in Figures 5H–H″, showing Prto-GFP **(E)**, Stv-V5 **(E′)**, and the merged image **(E′′)**. Colocalisation is observed in puctuae (arrowheads). Scale bar 20 µm. **(F–F′)** Localisation of the GFP tagged Cheerio 90KD isoform (F, green) and FLAG-tagged Prto peptides (**(F′)**, red) in muscles. Note that the striped pattern of Prto along Z-disks is narrower but coincides with Cher distribution in sarcomeres (arrowheads) **(F′′)**. Scale bar 20 µm.

SUPPLEMENTARY FIGURE S7
*prto-RNAi* knockdown with different Gal4 drivers provokes a reduction of adult wing size. Female (F) and male (M) adult wings of control (left panel) and *prto* KD (right panel). The whole wing area measurements for each KD condition are displayed graphically. The phenotypic analyses for *prto* KD are for Dlmo-Gal4 (**(A,B)**; top panel), 69B-Gal4 (**(C,D)**; middle panel) and en-Gal4 (**(E,F)**; bottom one) drivers. 1way ANOVA with Bonferroni post test statistics were performed. *n* = 11–49. Error bars represent 95% CI, *** = *p* < 0.0001, ** = *p* < 0.001, * = *p* < 0.05. Scale bar (red) 1000 µm.

SUPPLEMENTARY FIGURE S8
*prto* KD induces a reduction in wing cell number without affecting apoptosis and enhances *stv* mutant wing phenotypes. **(A)** Wild-type wing blade depicting identical size squares (highlighted with shadows) in the anterior compartment (2 squares at top in a horizontal array) and posterior compartment (2 squares near wing cross vein in a vertical arrangement). Number of wing cells in each compartment were calculated by averaging the number of trichomes in each square per compartment and extrapolating to the total area of each compartment. **(B)** Graph depicting the number of wing cells in anterior and posterior compartment showing that the posterior compartment of *prto* KD contain less cells than controls. Unpaired t-test were performed between *control* and *prto-RNAi* in anterior and posterior compartments. Error bars represent 95% CI, *** = *p* < 0.0001 **(C)**. *pacman (pcm)* ^*14*^ mutant wing imaginal discs (positive control) (Waldron et al., 2015) showing Caspase 3 (green) staining in the middle of the pouch (arrows). Scale bar 60 µm. **(D–D‴)** Control wing imaginal discs showing the merged image (D), Caspase 3 staining (**(D′)**; green), *en>UAS-dsRed;UAS-Dcr* expression (**(D′)**; red) and DAPI staining in nuclei (**(D‴)**; blue), scale bar 60 µm **(E)**. Graph showing the relative number of wing imaginal disc cells numbers expressing the NLS-dCycB-mRFP and dE2F1-GFP cell cycle markers in control and *prto* KD measured by the DeadEasy Mito-Glia program. Two tailed t-test was performed. Error bars denote 95% CI, * = *p* < 0.05. Note that the number of cells expressing both markers is increased in *en>UAS-Dcr;prto-RNAi* KDs. **(F–F‴)** Caspase 3 staining in *en>UAS-dsRed;UAS-Dcr,prto-RNAi* wing discs labelled as **(D)**, showing no evidence of Caspase 3 activation. **(G–I)** Male adult wings of *UAS-Dcr;prto-RNAi/stv* ^*1*^ controls **(G)**, *en>UAS-Dcr;prto-RNAi/MKRS* **(H)** and *en>UAS-Dcr;prto-RNAi/stv* ^*1*^ **(I)**. Scale bar 1 mm **(J)**. Graph showing the compartment area measurements. 1way ANOVA with Bonferroni post test was performed. *n* = 24–29, error bars represent 95% CI, *** = *p* < 0.0001, ** = *p* < 0.001, * = *p* < 0.05. Note that reducing the *stv* gene dosage enhances the wing are phenotype compared to *prto-RNAi* siblings, but no effect is observed in the anterior compartment.
