# Supplementary figures and images for: Purriato is a conserved small open reading frame gene that interacts with the CASA pathway to regulate muscle homeostasis and epithelial tissue growth in Drosophila
Source: Front Cell Dev Biol. 2023 Mar 10;11:1117454. doi: 10.3389/fcell.2023.1117454 (PMC10036370; doi:10.3389/fcell.2023.1117454)

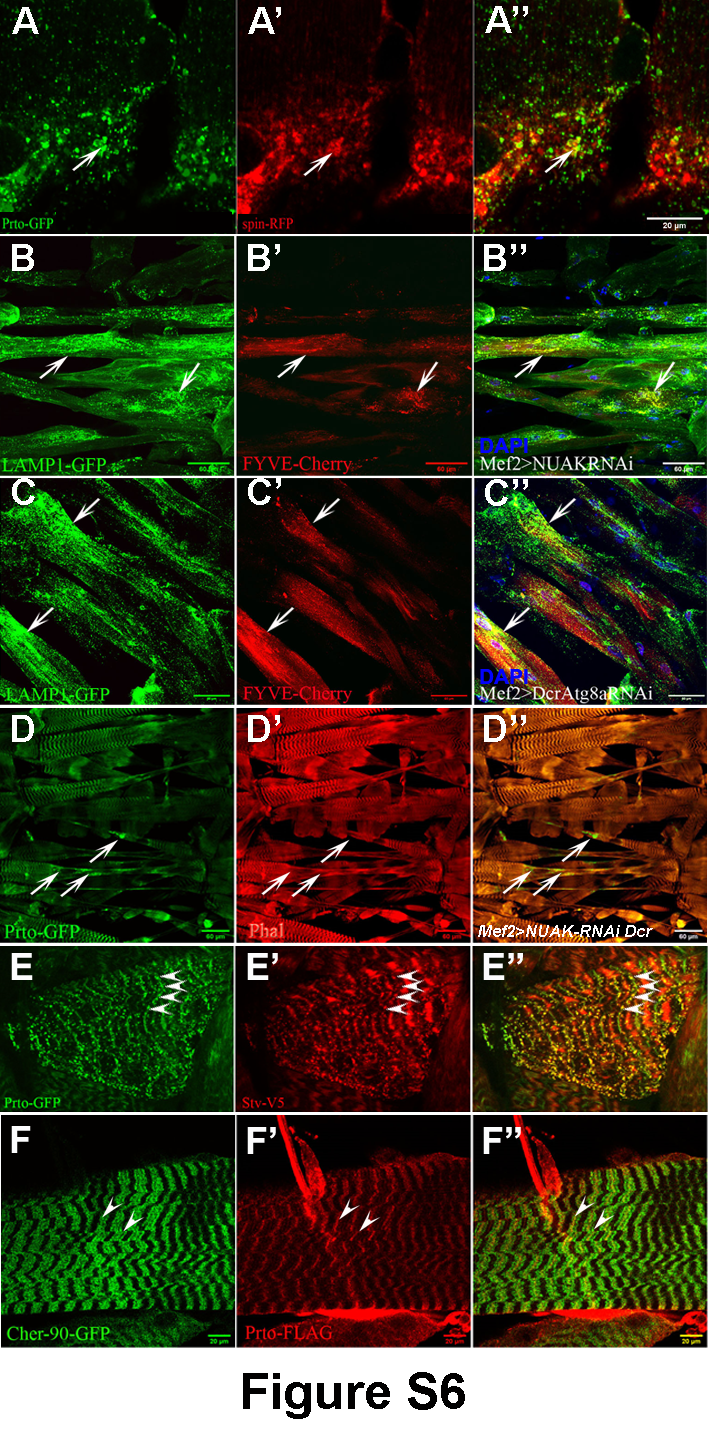

Supplement: Supplementary file 2 [file Image6.TIF]

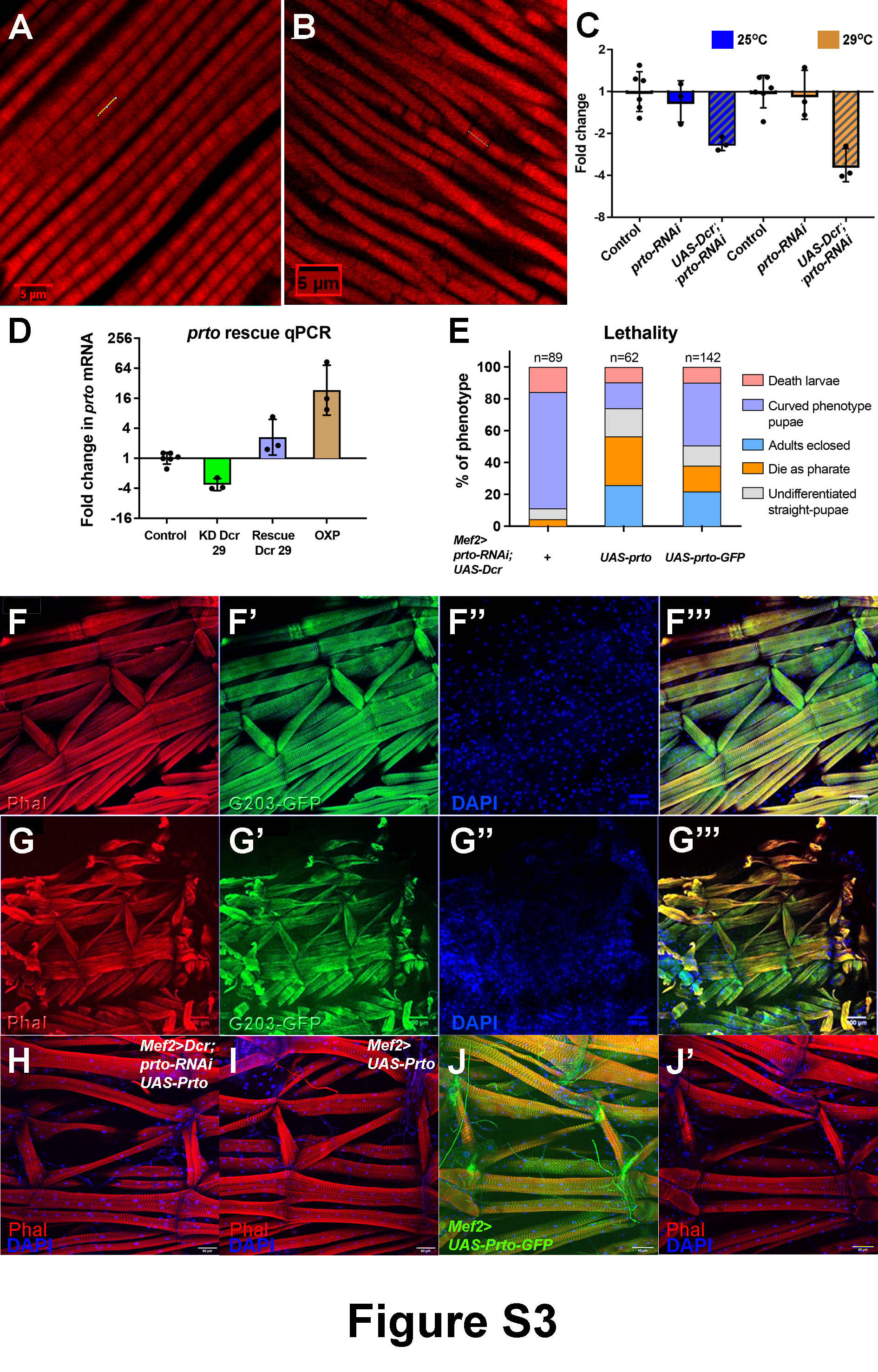

Supplement: Supplementary file 4 [file Image3.TIF]

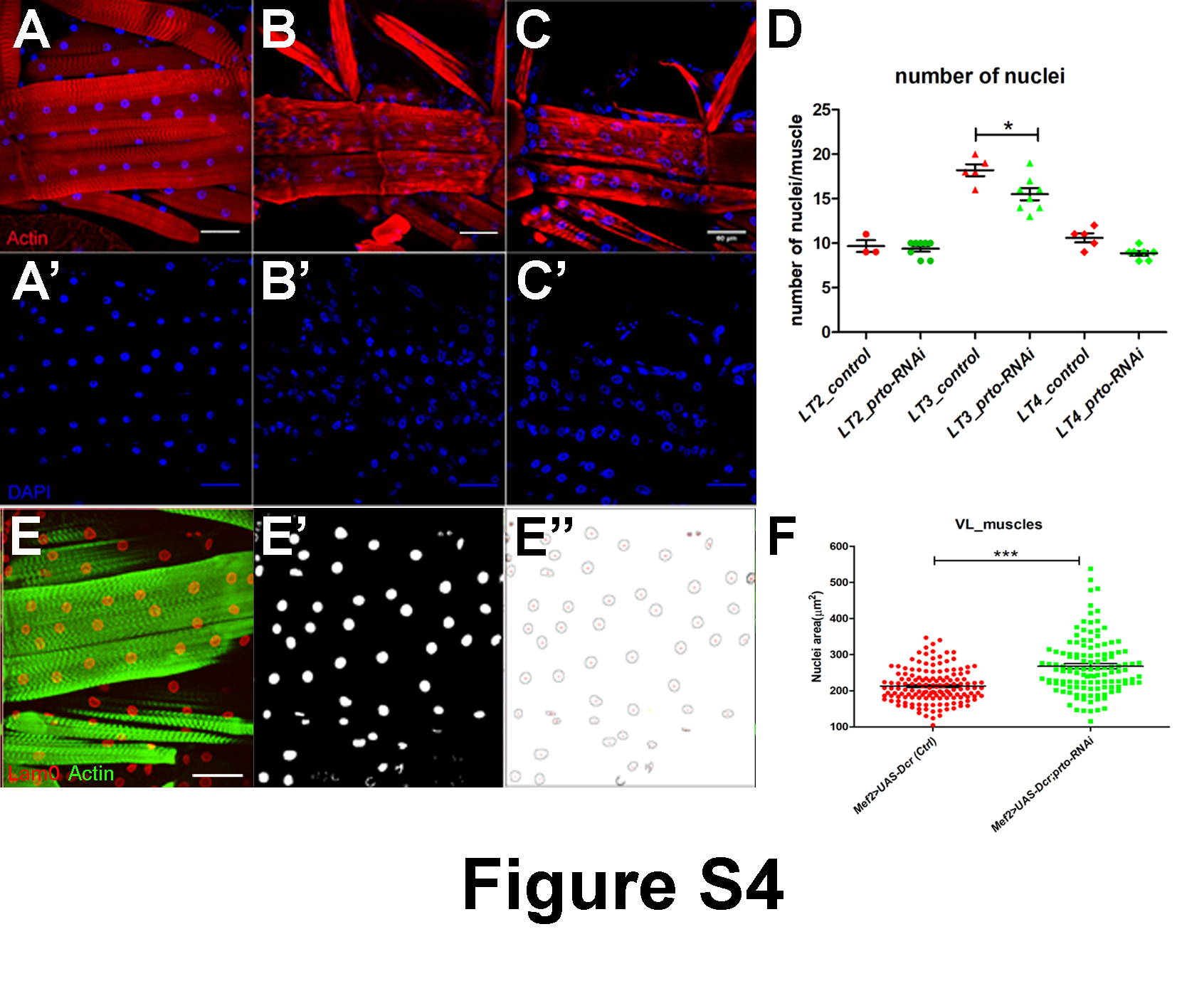

Supplement: Supplementary file 5 [file Image4.TIF]

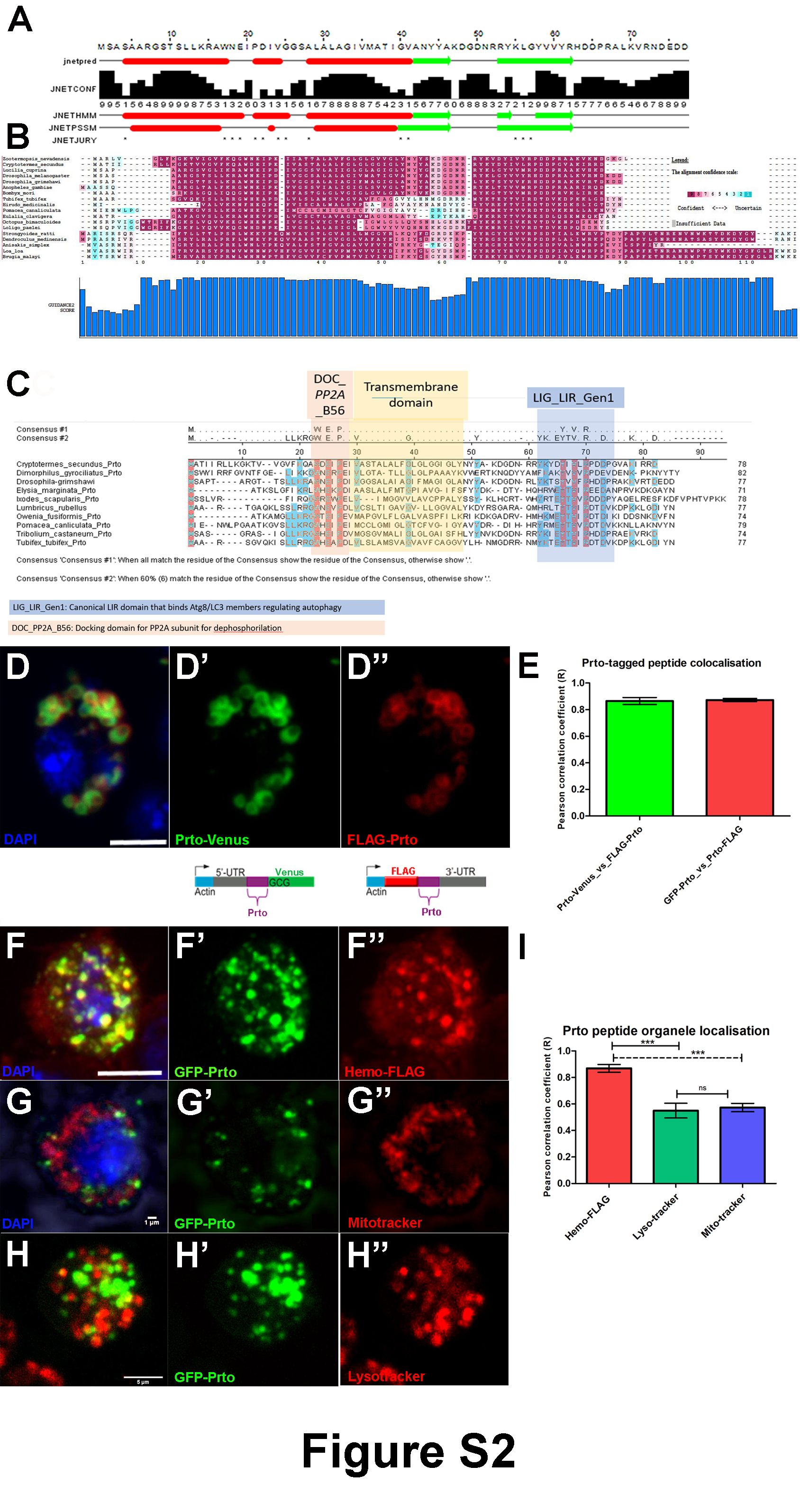

Supplement: Supplementary file 6 [file Image2.tif]

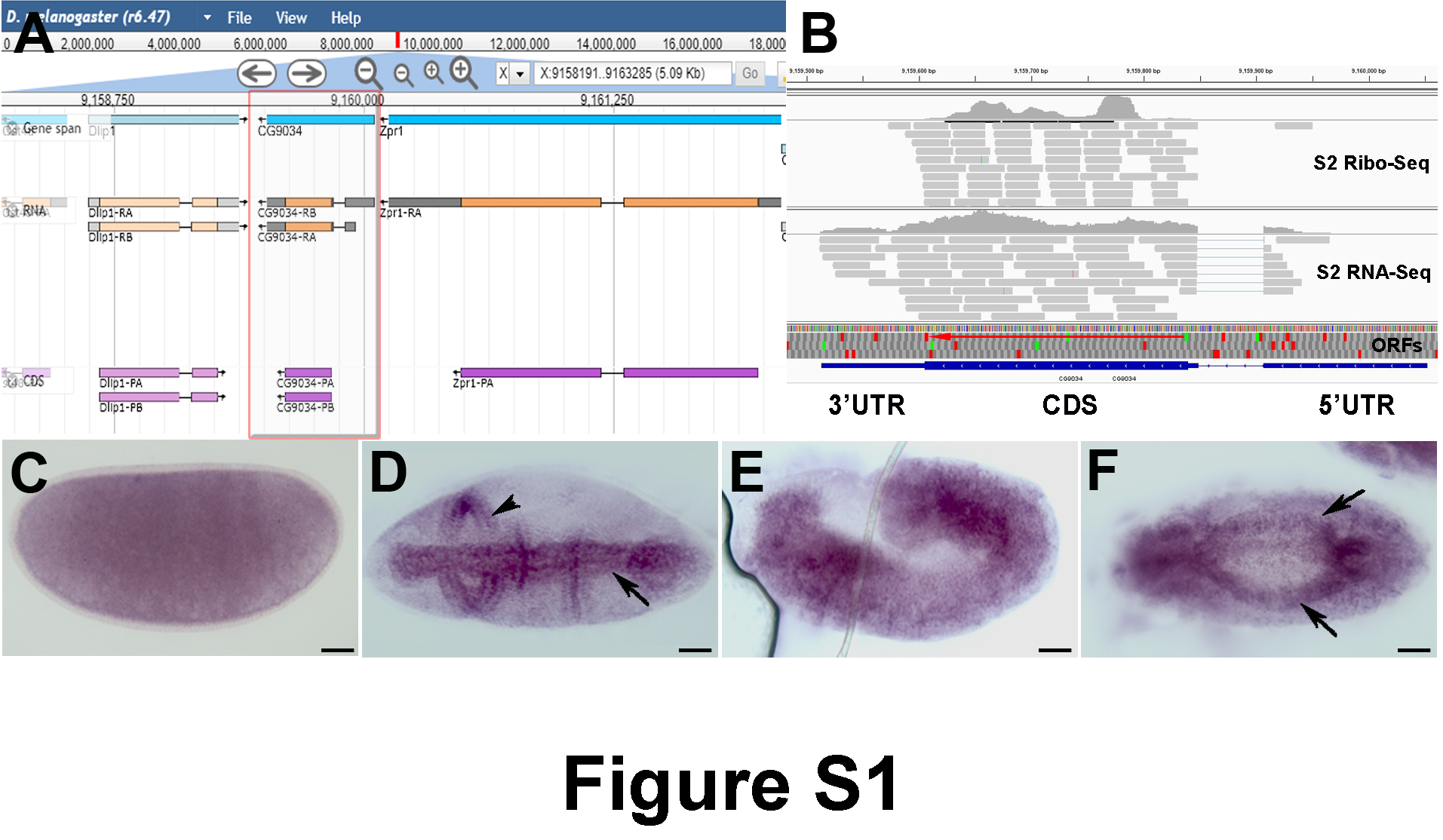

Supplement: Supplementary file 7 [file Image1.TIF]

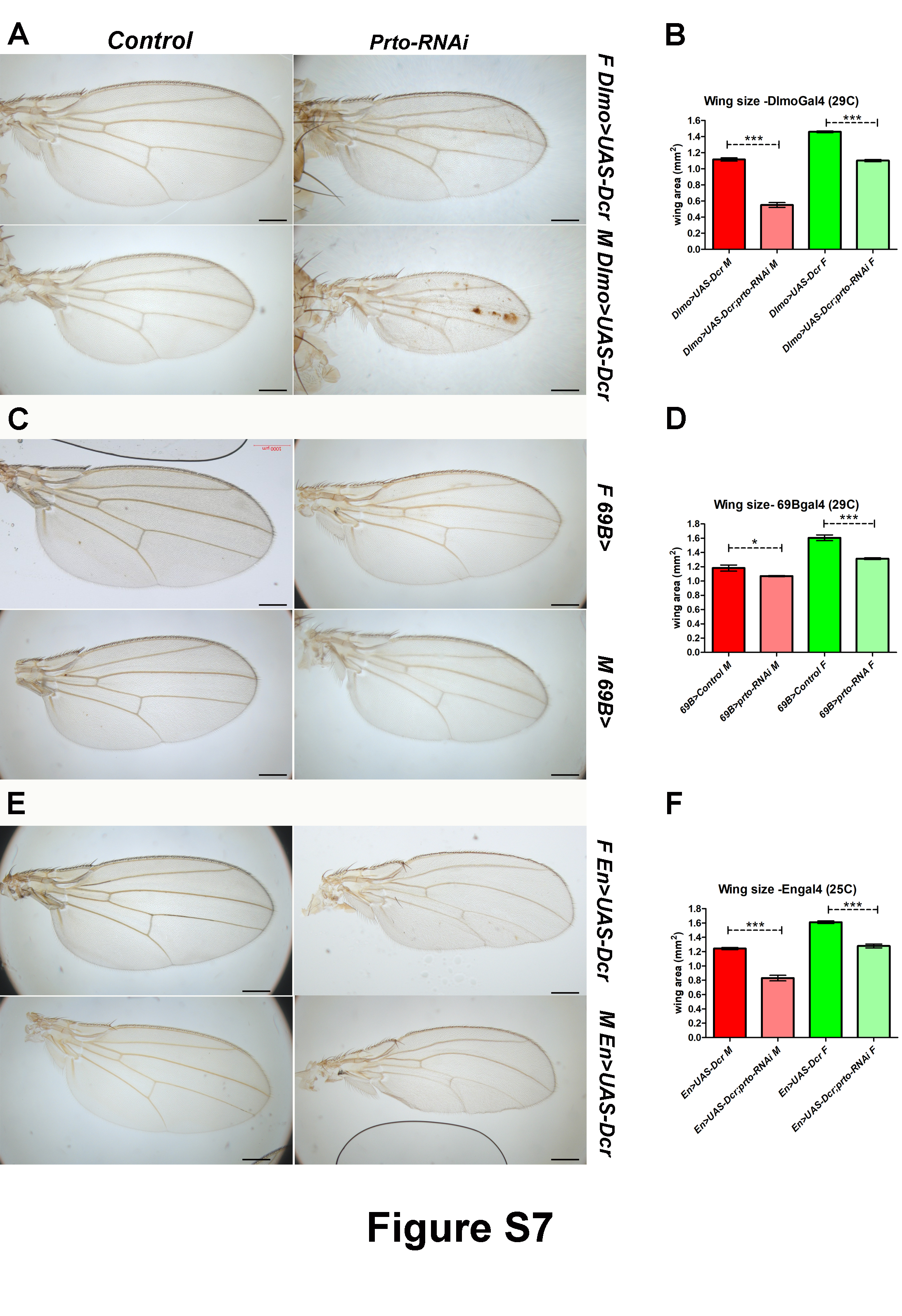

Supplement: Supplementary file 8 [file Image7.TIF]

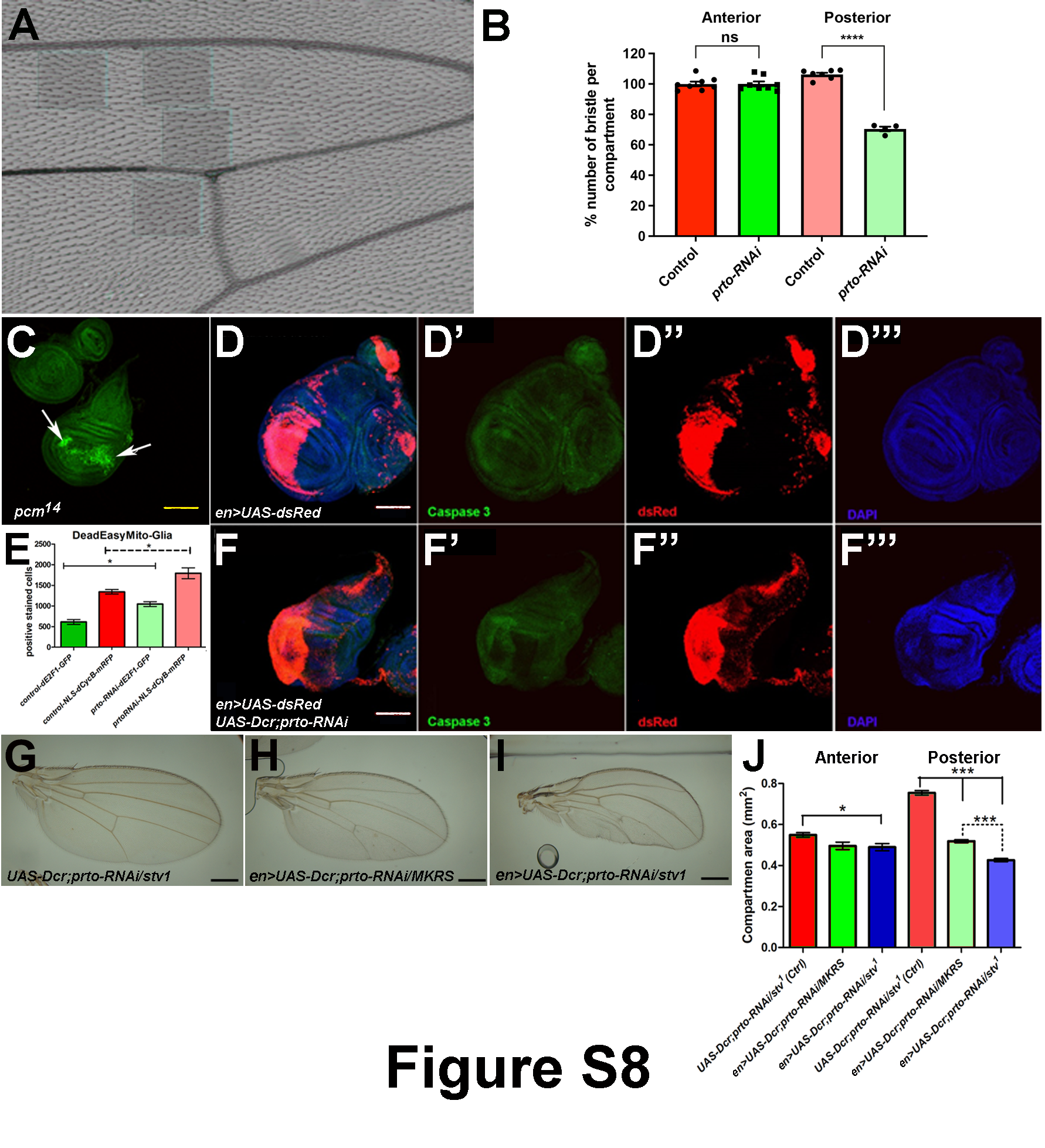

Supplement: Supplementary file 9 [file Image8.TIF]

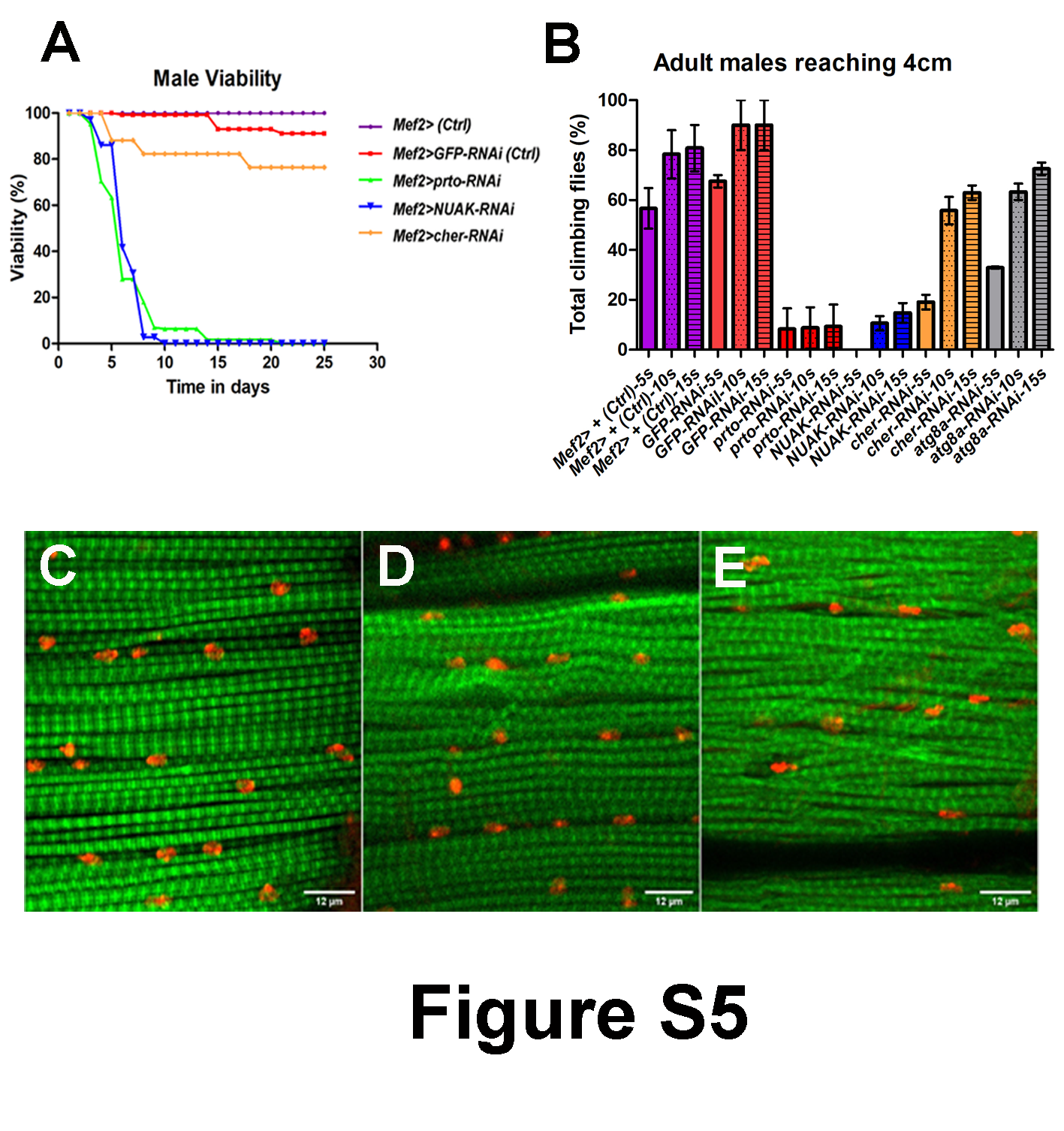

Supplement: Supplementary file 10 [file Image5.TIF]
