# Supplementary material for: Purriato is a conserved small open reading frame gene that interacts with the CASA pathway to regulate muscle homeostasis and epithelial tissue growth in Drosophila
Source: Front Cell Dev Biol. 2023 Mar 10;11:1117454. doi: 10.3389/fcell.2023.1117454 (PMC10036370; doi:10.3389/fcell.2023.1117454)
Supplement: Supplementary file 3 [file Table1.DOCX]

| Comparison |  | p-value | Fold change |
| --- | --- | --- | --- |
| Control vs *prto-RNAi* 25⁰C |  | 0.5107 | -1.22 |
| Control vs *UAS-Dcr*; *prto-RNAi* 25⁰C |  | 0.036 | -2.53 |
| Control vs *prto-RNAi* 29⁰C |  | 0.8194 | 1 |
| Control vs *UAS-Dcr*; *prto-RNAi* 29⁰C |  | 0.0004 | -2.75 |
| Control vs *UAS-Dcr*; *prto-RNAi; UAS-prto* 29⁰C |  | 0.0272 | 3.37 |
| Control vs *UAS-prto* 29⁰C |  | 0.0003 | 36.78 |

**Supplemental Table 1:** Summary of statistical information when assessing *prto* mRNA expression in larval carcasses following genetic manipulation. p-value calculated using a t-test for each comparison stated.
